# Transcriptome analysis suggests a compensatory role of the cofactors coenzyme A and NAD^+^ in medium-chain acyl-CoA dehydrogenase knockout mice

**DOI:** 10.1038/s41598-019-50758-0

**Published:** 2019-10-10

**Authors:** Anne-Claire M. F. Martines, Albert Gerding, Sarah Stolle, Marcel A. Vieira-Lara, Justina C. Wolters, Angelika Jurdzinski, Laura Bongiovanni, Alain de Bruin, Pieter van der Vlies, Gerben van der Vries, Vincent W. Bloks, Terry G. J. Derks, Dirk-Jan Reijngoud, Barbara M. Bakker

**Affiliations:** 1 Department of Pediatrics, University of Groningen, University Medical Center Groningen, Groningen, The Netherlands; 2 Department of Laboratory Medicine, University of Groningen, University Medical Center Groningen, Groningen, The Netherlands; 3 Department of Pathobiology, Faculty of Veterinary Medicine, Dutch Molecular Pathology Center, Utrecht University, Utrecht, The Netherlands; 4 HZPC Research B.V., Metslawier, The Netherlands; 5 Department of Genetics, University of Groningen, University Medical Center Groningen, Groningen, The Netherlands; 6 Genomics Coordination Center, University of Groningen, University Medical Center Groningen, Groningen, The Netherlands

**Keywords:** Proteomics, Transcriptomics, Metabolic disorders, Translational research, Pathogenesis

## Abstract

During fasting, mitochondrial fatty-acid β-oxidation (mFAO) is essential for the generation of glucose by the liver. Children with a loss-of-function deficiency in the mFAO enzyme medium-chain acyl-Coenzyme A dehydrogenase (MCAD) are at serious risk of life-threatening low blood glucose levels during fasting in combination with intercurrent disease. However, a subset of these children remains asymptomatic throughout life. In MCAD-deficient (MCAD-KO) mice, glucose levels are similar to those of wild-type (WT) mice, even during fasting. We investigated if metabolic adaptations in the liver may underlie the robustness of this KO mouse. WT and KO mice were given a high- or low-fat diet and subsequently fasted. We analyzed histology, mitochondrial function, targeted mitochondrial proteomics, and transcriptome in liver tissue. Loss of MCAD led to a decreased capacity to oxidize octanoyl-CoA. This was not compensated for by altered protein levels of the short- and long-chain isoenzymes SCAD and LCAD. In the transcriptome, we identified subtle adaptations in the expression of genes encoding enzymes catalyzing CoA- and NAD(P)(H)-involving reactions and of genes involved in detoxification mechanisms. We discuss how these processes may contribute to robustness in MCAD-KO mice and potentially also in asymptomatic human subjects with a complete loss of MCAD activity.

## Introduction

Hepatic mitochondrial fatty-acid oxidation (mFAO) is important for the generation of ATP and ketone bodies during fasting, and indirectly also for *de novo* synthesis of glucose by providing the required ATP. Children with a loss-of-function c.985A > G mutation in the *ACADM* gene, encoding the mFAO enzyme medium-chain acyl-CoA dehydrogenase (MCAD), run a severe risk of life-threatening hypoglycemia^1,2^. Nevertheless, a subset of these children never develop any symptoms^3–11^. Notably, under resting conditions MCAD-deficient children oxidize medium-chain fatty acids normally^12^ and they are able to fast for 18–24 hours^2,3,13,14^. Particularly a combination of prolonged fasting and an additional trigger, such as infections, increases the risk of low blood glucose levels^1,11,15–17^.

The mFAO pathway is schematically depicted in Supplementary Fig. 1. Fatty acids are taken up into the mitochondria in an activated form as acyl-CoA esters and subsequently oxidized to acetyl-CoA. This occurs in repetitive four-reaction cycles. In each cycle, the acyl-CoA ester is shortened by two carbon atoms, thereby producing acetyl-CoA^2,18,19^. The first reaction in each cycle is catalyzed by a set of isoenzymes, which accept a range of acyl-CoA substrates of different, overlapping carbon-chain lengths. In rodents, these are very-long-, long-, medium-, and short-chain acyl-CoA dehydrogenase (VLCAD, LCAD, MCAD, and SCAD). In humans, the LCAD protein concentration is low in the liver and plays a minor role in the mFAO pathway^2,20^. The coenzymes nicotinamide adenine dinucleotide (NAD^+^) and free CoA (CoASH) play key roles in mFAO function and link the pathway to other metabolic pathways^21–25^.

To investigate the disease etiology in MCAD deficiency (MCADD), an MCAD-KO mouse model was generated, initially in a C57BL/6NTac & 129P2/OlaHsd mixed background^26^. The mouse recapitulated a number of human disease characteristics, including elevated neonatal death rate, lower C8-acyl-CoA oxidation capacity, higher urinary medium-chain organic acid levels, and higher serum levels of medium-chain acyl-carnitine levels compared to the wild type (WT). In addition, 24 of hours fasting induced lipid accumulation (steatosis) in the liver in the MCAD-KO but not the WT mice, in accordance to hepatic steatosis seen in symptomatic MCADD patients^1,11,15–17^. Finally, when fasted in combination with cold-exposure, the MCAD-KO mouse showed significantly lower body temperature compared to WT, which was eventually lethal^26^. Hepatic microarray analysis showed marked differences in the expression of glucose metabolism genes between fasted WT and KO mice^27^. In the meantime, we backcrossed the MCAD-KO mouse model to a pure C57BL/6J background^20^. In contrast to the mixed background, this allows comparison to a monogenetic wild-type control. The C57BL/6J was chosen because it is widely used and many data are available for comparison. Like its predecessor, the pure-background MCAD-KO mouse exhibited higher medium-chain acyl-carnitine levels in serum and a lower C8-acyl-CoA oxidation capacity compared to the WT after 12 hours of fasting^20^. Notably, the low blood glucose levels which are the hallmark of human MCADD, were never reported in MCAD-KO mice^26–28^. Therefore, the MCAD-KO mouse has so far not been a useful model for the acute hypoglycemia observed in symptomatic MCADD patients. It may, however, be an excellent model to identify potential compensatory mechanisms to explain why some humans remain asymptomatic despite a homozygous loss-of-function mutation in the *Acadm* gene that encodes the MCAD enzyme. This may help to identify patients who are not at risk and are currently overtreated^29^ and provide clues for prevention in patients who are still at risk.

The aim of the current study is to explore hepatic functional and molecular differences between WT and MCAD-KO mouse to gain insight into the role of the liver in these compensatory mechanisms. To this end we subjected the C57BL/6J pure-background MCAD-KO mouse model to low-fat and high-fat diet, and studied them under fed and 16 h fasted conditions. The high-fat diet is new compared to previously published studies and may provide an additional environmental perturbation to the MCAD-KO mouse by providing the mFAO pathway with additional substrate. We collected biometric, histological, targeted proteomics, and transcriptomics data from liver tissue. In addition, hepatic mitochondrial function was analyzed. Pattern recognition analysis showed subtle adaptations in detoxification processes and in genes coding for enzymes involved in CoA and NAD(P)(H) metabolism of MCAD-KO mice. We discuss how these adaptations may contribute to their phenotypic robustness.

## Results

### Biometric and hepatohistological characterization of the pure-background MCAD-KO mice

MCAD-KO and WT C57BL/6J mice were kept on a low-fat control diet or a high-fat semisynthetic diet for 6 weeks and subsequently fasted for 16 hours. This 6 weeks treatment minimized secondary effects of the diet, such as obesity. In addition, MCADD patients are most vulnerable during infancy, when they are more dependent on a high mFAO capacity. Therefore, relatively young mice (2–4 months) were used.

**Figure 1:**
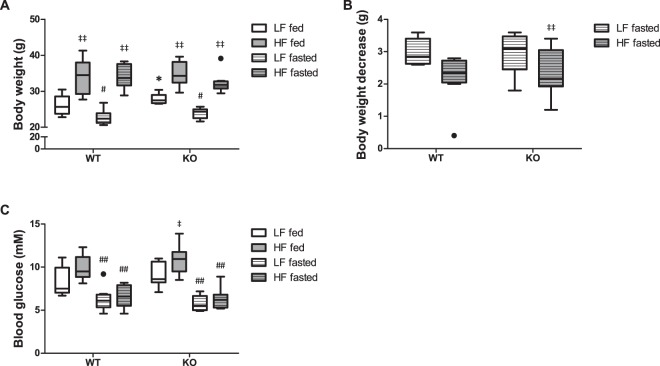
Mouse biometric measures (**A–C**) under different conditions (**A**) Body weight; (**B**) body weight decrease after fasting; (**C**) Blood glucose levels at termination. The results are represented as Tukey box and whisker plots, in which the black-filled circles indicate individual mice falling outside the 75% percentile plus 1.5·inter-quartile range (IQR) or 25% percentile minus 1.5·IQR. n = 6–8 for both WT and KO. *p < 0.05 compared to WT, ^#^and ^##^p < 0.05 and p < 0.01 compared to fed, respectively, ^‡^and ^‡‡^p < 0.05 and p < 0.01 compared to low-fat diet (LF), respectively.

Little to no effect of genotype was observed on body weight, body-weight loss during fasting, liver weight, or blood glucose level (Figs 1A–C and S2,A,B). Only in the low-fat fed group the body weight was statistically higher in MCAD-KO compared to WT mice (Fig. 1A), but the difference was only 8%. These results are in accordance with earlier studies^26–28^.

**Figure 2:**
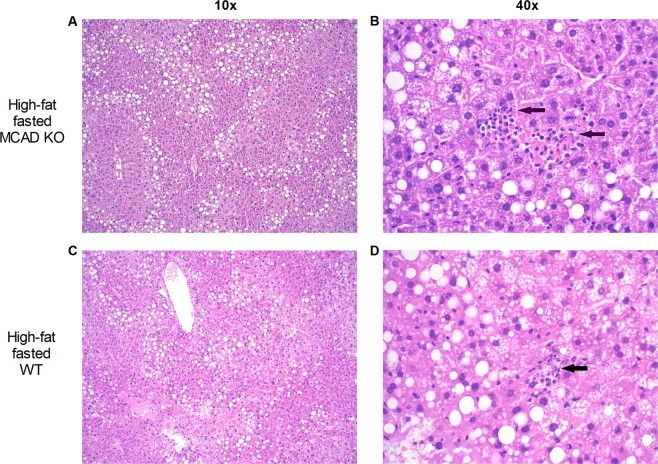
Similar steatosis grade (**A,C**) and difference in lobular inflammation (**B,D**) between MCAD-KO and WT mice in the high-fat fasted condition with inflammatory loci shown by the black arrows. Shown here are representative pictures. Livers were stained by Hematoxylin & Eosin (see Materials and Methods for further details). Inflammation is identified by infiltration of leukocytes. Lobular inflammation was recognized as small groups of mixed inflammatory cells scattered throughout the hepatic parenchyma (see Supplementary Table ST1 for semi-quantitative scoring of the livers). n = 6–8 for both WT and KO.

Since hepatic steatosis had been reported after fasting of the mixed-background MCAD-KO mouse^26^, we inspected the liver in more detail. Histological examination showed that there were no statistically significant differences between WT and KO (Figs 2 and S3, S4). However, the MCAD-KO mice did show a trend towards a higher degree of lobular inflammation in the high-fat fasted condition (Fig. 2 and Supplementary Table ST1). Liver triglyceride concentrations, however, were not significantly different in MCAD-KO compared to WT mice under any of the conditions (Supplementary Fig. S2C).

### MCAD-KO mice show decreased C8-acyl-CoA-dependent state 3 O_2_ consumption capacity

**Figure 3:**
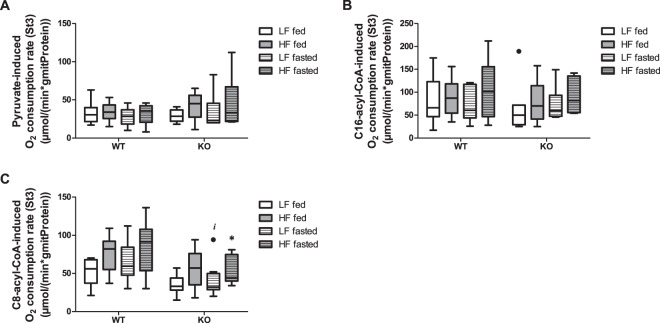
Mouse hepatic mitochondrial function in different conditions. Pyruvate- (**A**), C16-acyl-CoA- (**B**) and C8-acyl-CoA- (**C**) induced maximum O2-consumption flux (state 3) in isolated liver mitochondria. State 3 represents the maximum ADP-stimulated oxygen consumption. The results are represented as Tukey box and whisker plots, in which the black-filled circles indicate individual mice falling outside the 75% percentile plus 1.5·inter-quartile range (IQR) or 25% percentile minus 1.5·IQR. n = 6–8 for both WT and KO. *p < 0.05 compared to WT, ^#^and ^##^p < 0.05 and p < 0.01 compared to fed, respectively, ^‡^and ^‡‡^p < 0.05 and p < 0.01 compared to low-fat diet (LF), respectively.

We confirmed the primary biochemical defect in MCAD function by measuring the substrate-dependent O_2_-consumption rate in isolated liver mitochondria (Figs 3 and S5). On pyruvate or C16-acyl-CoA as substrates, the genotype had no effect on O_2_-consumption rate (Fig. 3A,B), in line with expectations and earlier results^20^. Previously, more detailed analysis showed that C8-acyl-carnitine accumulated in isolated mitochondrial preparations with C16-acyl-CoA as a substrate^20^. The MCAD-KO mouse had a lower O_2_ consumption rate than the WT on the MCAD substrate C8-acyl-CoA (Fig. 3C), again consistent with our earlier results^20^. This was significant in the high-fat diet fasted group (p = 0.02) and statistically indicative in the low-fat diet fasted group (p = 0.06) (with the average MCAD-KO rates being 72 and 62% of the average WT rates on low-fat and high-fat diet, respectively). Also under fed conditions the C8-acyl-CoA-dependent O_2_-consumption rate was lower in the MCAD-KO (with the average MCAD-KO rates being 65% and 74% of the average WT rates on low-fat and high-fat diet, respectively), but this difference was not statistically significant (p = 0.15 and 0.13, respectively). The fact that there is such a high residual C8-acyl-CoA-dependent O_2_-consumption rate in the MCAD-KO mouse may be attributed to the isoenzymes SCAD and LCAD, which are known to be expressed in mouse liver^28^. In the following section, we will investigate the protein concentrations of these and other mitochondrial enzymes in more detail.

### Targeted mitochondrial proteomics

**Figure 4:**
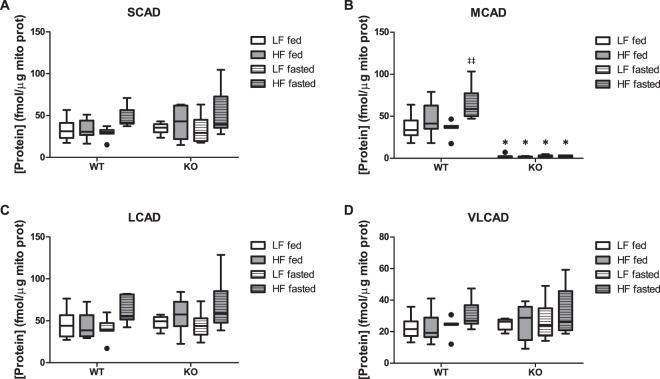
Absolute protein levels of the mFAO proteins SCAD (**A**), MCAD (**B**), LCAD (**C**) and VLCAD (**D**). The other mitochondrial energy metabolism proteins are shown in Supplementary Fig. S6. The results are represented as Tukey box and whisker plots, where the black-filled circles indicate individual mice falling outside the 75% percentile plus 1.5·inter-quartile range (IQR) or 25% percentile minus 1.5·IQR. n = 6–8 for both WT and KO. *p < 0.05 compared to WT, ^#^and ^##^p < 0.05 and p < 0.01 compared to fed, respectively, ^‡^and ^‡‡^p < 0.05 and p < 0.01 compared to low-fat diet (LF), respectively.

Recently, we developed a targeted proteomics method to detect and quantify specific proteins involved in mitochondrial energy metabolism. The targeted proteins comprise all enzymes involved in mFAO and TCA cycle, core subunits of each of the respiratory-chain complexes and metabolite transporters required for these pathways^28^. We applied this method to the hepatic mitochondria isolated in this study (Figs 4A–D and S6). As expected, the MCAD protein was not detectable in the MCAD-KO mitochondria. The isoenzymes SCAD and LCAD, which also accept MCAD substrates, were expressed (Fig. 4A,C) and they could thus explain the presence of a residual flux of C8-acyl-CoA oxidation discussed above. However, neither their expression, nor that of the other targeted proteins were altered in the MCAD-KO mouse compared to the WT (Supplementary Fig. S6). Thus, the targeted proteomic analysis did not identify any compensatory mechanisms involving the concentration of the measured proteins. Since the proteomics was done on isolated mitochondria, we measured citrate synthase activity in liver homogenates as a proxy for mitochondrial content (Supplementary Fig. S7). No significant difference between WT and MCAD-KO was observed.

### Differences in hepatic mRNA expression patterns between MCAD-KO and WT mouse

**Figure 5:**
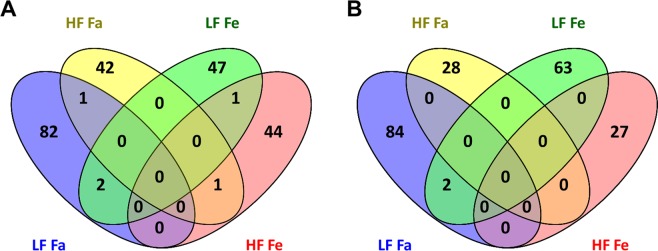
Venn diagrams showing overlap between conditions of upregulated (**A**) and downregulated (**B**) genes between WT and KO for a p < 0.01. The depicted results are based on the DeSeq2 method.

To explain the physiological robustness of the MCAD-KO mice, we analyzed the liver transcriptome by RNAseq. Principle component analysis showed that fasting had a much stronger effect on the transcriptome than the genotype (Supplementary Fig. S8). Through differential gene expression (DGE) analysis, we calculated differences in transcript levels between MCAD-KO and WT mice. A relatively small number of transcripts were affected by the genotype according to the exploratory p-value of <0.01 and the corresponding fold changes were predominantly between 1 and 2 (Supplementary Fig. S9A–D). In addition, the genes in question showed little overlap between the conditions (Figs 5A,B and S9E,F). Notably, four transcripts were identified as significantly altered by the genotype according to p_adj_ < 0.05; three transcripts for the low-fat fasted condition and one for the high-fat fed condition, respectively (Supplementary Table ST2). Interestingly, *Akr1b7*, a gene coding for Aldose reductase-related protein 1, an enzyme that uses NADP^+^, was identified under the low-fat fasted condition. *Acnat2*, which codes for acyl-coenzyme A amino acid N-acyltransferase 2, an enzyme that produces CoA, was identified under the high-fat fed conditions. As metabolic gene expression changes can be small and to nevertheless identify functional metabolic differences between the KO and WT, pathway analysis may be more insightful.

#### Gene-set enrichment analysis revealed differentially expressed gene sets between WT and KO mice

Gene-set enrichment analysis (GSEA) determines whether the members of predefined sets of functionally related genes are predominantly found at the top or bottom of the list of genes ranked according to the t-value corresponding to their differential expression. We applied GSEA with a focus on metabolism. To this end we used a metabolic gene-set collection consisting of the KEGG (Kyoto Encyclopedia of Genes and Genomes) metabolic gene set collection complemented with sets coding for enzymes that have CoASH or NAD(P)(H) as a substrate or product, respectively (Supplementary Table ST3). In the low-fat fasted and high-fat fed condition no metabolic gene set was up- or downregulated compared to WT with a false discovery rate (FDR) of <0.25. In the low-fat fed condition 2 gene sets were upregulated and 10 were downregulated (Table 1). In the high-fat fasted condition 8 gene sets were downregulated. In the low-fat fed condition, the “Pyruvate metabolism” and “Fatty-acid biosynthesis” gene sets were significantly downregulated (FDR < 0.05, Table 1). The other gene sets with an FDR of <0.25 suggest a potential downregulation of lipid-related metabolism (see underlined gene sets in Table 1). Zooming into these gene sets, we observed that in 6 out of the 12 gene sets, the genes involved in CoA and NAD(P)(H) metabolism were overrepresented among the differentially regulated genes, as quantified by Risk Ratios (Table 1 and Supplementary Tables 4, 5).

**Table 1:** Up- and downregulated metabolic gene sets in KO compared to WT based on the GSEA method.

*Upregulated gene sets*			
**LF Fed**			
Metabolism of xenobiotics by cytochrome P450	*		
Taurine and hypotaurine metabolism	*		
***Downregulated gene sets***			
**LF Fed**		**HF Fasted**	
**Pyruvate metabolism**		**Metabolism of xenobiotics by cytochrome p450**	
**Fatty acid biosynthesis**		**Drug metabolism - cytochrome p450**	
**Sulfur metabolism**		**Glutathione metabolism**	
Histidine metabolism		NAD(P)(H) main	
Synthesis and degradation of ketone bodies	*	Steroid hormone biosynthesis	*
Other types of o-glycan biosynthesis		NAD(P)(H) redox	
Biosynthesis of unsaturated fatty acids	*	Oxidative phosphorylation	*
Fatty acid metabolism	*	Taurine and hypotaurine metabolism	
Citrate cycle (TCA cycle)	*		
Glycosphingolipid biosynthesis - lacto and neolacto series			

Downregulated and upregulated gene sets comprise gene sets that are downregulated and upregulated, respectively, in KO compared to WT. Gene sets depicted here have an FDR of <0.25 (explorative) and gene sets in bold have an FDR of <0.05 (statistically significant). *Gene sets in which CoA and NAD(P)(H) genes are overrepresented in the list of contributing genes. Underlined are lipid-metabolism-related gene sets. The exact risk ratios and the list of contributing genes are given in ST7–9.

In the high-fat fasted condition, metabolism of xenobiotics by cytochrome p450, drug metabolism – cytochrome p450, and glutathione metabolism were significantly downregulated (FDR <0.05). Notably, these three gene sets are all related to detoxification. In the wider collection of downregulated gene sets (FDR <0.25) we also found that CoA- and NAD(P)H-related genes were substantially overrepresented among the genes that contributed to the downregulated gene sets. (Tables 1 and ST6).

We also performed GSEA on the microarray dataset of fed and 12-hr-fasted MCAD-KO and WT mice from Herrema *et al*.^27^, as described in Materials and Methods. Four gene sets were upregulated and 8 were downregulated, all with an FDR between 0.05 and 0.25 (Supplementary Table ST7). The results confirm the gene set enrichments in lipid metabolism and pyruvate metabolism found in our study. Zooming into the 12 gene sets, we found that in 4 out of the 7 gene sets that contain CoA and/or NAD(P)(H) genes (other than the CoA gene set itself), the genes involved in CoA/NAD(P)(H) metabolism were overrepresented among the differentially regulated genes (Supplementary Tables ST8, 9). In summary, GSEA suggested that there was a limited metabolic compensation for the loss of MCAD activity. However, CoASH- and NAD(P)(H)-dependent reactions were overrepresented.

#### Alternative gene set enrichment analysis reveals a role for CoA and NAD(P)(H) metabolism

When searching for mechanisms that compensate for the loss of MCAD activity under multiple conditions, standard GSEA has a limitation. It only identifies gene sets that either contain predominantly upregulated or predominantly downregulated genes, whereas a mixture of up- and downregulated genes in a single gene set may have a strong effect on metabolite concentrations. Therefore, we designed an alternative method to also identify gene sets which are substantially (over)represented in both directions. In addition, we subsequently also identified gene sets that are (over)represented under more than one condition. In short, we created a separate list of upregulated and a separate list of downregulated genes, for each condition (WT vs KO comparison; Supplementary Table ST10). We then ranked gene sets on the basis of their (over)representation in these lists. Subsequently, the gene sets were ranked based on their median ranking over all conditions (see Methods for details and Tables 2, ST11 and ST12 for results). The top 10 gene sets for each direction are shown in Table 2. Particularly NAD(P)(H)- and CoA gene sets were identified at the top of the list with an overrepresentation in both up- and downregulated genes. Moreover, purine metabolism appeared in the top ranking, overrepresented in up- and downregulated genes lists. Since the coenzymes CoASH and NAD(P)^+^ contain a purine building block, these three gene sets directly relate to each other. The lower-ranked gene sets predominantly suggest changes in lipid metabolism (underlined gene sets in Table 2). Together with pyruvate metabolism 6 of these 7 lipid metabolism gene sets belong to the top 50% of gene sets with the highest percentage of genes coding for enzymes that catalyze CoASH or NAD(P)(H) containing reactions (Supplementary Table ST13). Thus, the results of the alternative gene set enrichment analysis corroborate our conclusion that reactions involved in CoA- and NAD(P)(H) metabolism are markedly overrepresented among the differentially expressed genes in the MCAD-KO versus the WT, in both directions.

**Table 2:** Up- and downregulated metabolic gene sets in KO compared to WT based on the Alternative GSEA method.

Upregulated gene sets		Downregulated gene sets	
NAD(P)(H) main	*,^‡^	**NAD(P)(H) main**	*,^‡^
NAD(P)(H) redox	*,^‡^	**NAD(P)(H) redox**	*,^‡^
Purine metabolism	*,^‡^	CoA gene set	*,^‡^
CoA gene set	*,^‡^	Arachidonic acid metabolism	*,^‡^
Retinol metabolism	*	Purine metabolism	*,^‡^
Steroid hormone biosynthesis	*	***Pyruvate metabolism***	*,^‡^
Glycerolipid metabolism	*	Inositol phosphate metabolism	*
Fructose and mannose metabolism	*	Glycerophospholipid metabolism	*,^‡^
Galactose metabolism	*	Alanine, aspartate and glutamate metabolism	*
Pyrimidine metabolism	*	**Fatty acid metabolism**	*,^‡^

Depicted here are the top 10 gene sets with the most amount of differentially-expressed genes according to the “(|log2(fold change)| >0.5 AND p < 0.1) OR p < 0.05” criteria. *Amount of differentially expressed genes of the given pathway is overrepresented in 2 or more conditions in the given direction. ‡Overlap with the top 15 of the dataset of Herrema *et al*.^27^. Bold: Overlap with GSEA results for the Metabolic gene sets with FDR <25%. Bold and italic: Overlap with GSEA results for the Metabolic gene sets with FDR <5%. Underlined are lipid-metabolism-related gene sets. Additional results are given in Supplementary Tables ST10–13.

#### Validation: *Acot2* expression is elevated in MCAD-KO mice

To validate the RNASeq results, we first checked if the downregulated detoxification pathways affected lipid peroxidation. However, the thiobarbituric acid-reactive species (TBA-RS) assay, which measures malondialdehyde and is an indicator of lipid peroxidation, did not show a significant difference in plasma between WT and MCAD-KO mice (Supplementary Fig. S10).

**Figure 6:**
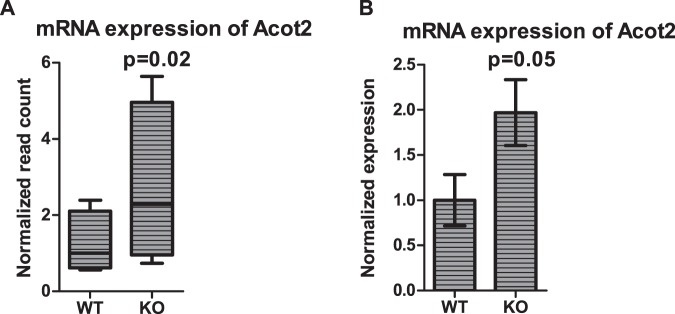
*Acot2* gene expression. *Acot2* gene expression in the LF fasted condition by RNASeq (**A**) and in LF fasted condition by real-time PCR (**B**). The RNASeq results are expressed as normalized read count relative the median normalized read count of the WT mice and are represented as Tukey box and whisker plots (n = 4). The real-time PCR results are expressed as *Acot2* expression relative to the average expression in the WT mice and are represented as bar plot (mean ± SEM) (n = 8).

Among the differentially expressed CoA-related genes, the acyl-CoA thioesterases (Acots) are known to stimulate mFAO^30–33^ and therefore provide a logical link to mFAO defects, such as MCADD. Therefore, we examined the expression of all 15 *Acot* genes^31,32^, for the high-fat and low-fat conditions. The expression of several of these genes was higher under fasted compared to fed conditions (Supplementary Fig. S11), in accordance with previous results^31^. This is compatible with a role of the ACOT enzymes to stimulate fatty-acid oxidation during fasting. More interestingly, the most upregulated mitochondrial *Acot* gene in the liver during fasting, *Acot2*, was even further upregulated in the MCAD-KO compared to WT mouse liver in the low-fat fasted state (RNASeq FC of 1.8 and p < 0.05; Fig. 6A) and was also the only gene that was differentially expressed between WT and KO in any of the fasted conditions. This higher expression was also confirmed by quantitative real-time PCR (FC of 2.0 and p = 0.05; Fig. 6B). These results suggest that extra stimulation of the mFAO by the *Acot2* mRNA during fasting may compensate for the limited mFAO capacity in the MCAD-KO mouse.

## Discussion

In this study, we aimed to explore the role of the liver in the MCADD phenotype in the C57BL/6J MCAD-KO mouse model. For this, we generated and analyzed a comprehensive physiological, histological and molecular dataset. The complete loss of MCAD protein led to a 30–40% reduction of the mitochondrial capacity for oxidation of the medium-chain substrate octanoyl-CoA. In principle, LCAD can carry the residual flux in mice, but it has a lower catalytic capacity and affinity for medium-chain substrates^20^. The concentration of LCAD and its short-chain isoenzyme SCAD was not altered in the MCAD-KO mice. In a previous study we have measured the ACAD activity on the acyl-CoA substrates of carbon-chain length C4, C6, C8, C10, C12, C14, and C16^20^. This gave only significant differences between WT and KO on C10-acyl-CoA, suggesting that SCAD, LCAD and VLCAD had no altered activity, despite potential regulation of their activity by acetylation^20^. Together, these results show that LCAD can only compensate partly for the loss of MCAD activity. Nevertheless, as also previously reported, the glucose levels and liver weight were similar between WT and MCAD-KO mice^26,27,34^. A trend towards a hepatic histopathological phenotype was observed in the MCAD-KO mice in the high-fat fasted condition, in agreement with earlier results^26^. This suggests hepatotoxicity as a result of previously observed medium-chain lipid accumulation in MCAD-KO mice^20,26,27^. Medium-chain lipids have been reported to cause oxidative damage^35–40^ and hepatic steatosis^41^, and to impair detoxification mechanisms^42,43^. Interestingly, the transcriptomic data suggest impaired detoxification mechanisms in the MCAD-KO mouse. We could not confirm altered lipid peroxidation in mouse plasma, but oxidative damage to lipids and proteins as well as decreased antioxidant capacity was reported in MCAD-deficient patients^44^. Humans already have less CYP4A and CYP4F genes than mice, which may explain why they are more sensitive. Moreover, the TBA-RS assay measures only malondialdehyde, whereas other lipid peroxidation products may be formed.

Classical and alternative GSEA of the transcriptomics data revealed CoASH and NAD(P)(H) as common denominators distinguishing MCAD-KO and WT mice. Both CoASH and NAD(P)(H) link mFAO to other pathways in metabolism^21–25^. It has been proposed that in deficiencies of CoA ester degradation, including MCAD deficiency, CoA may be sequestered in esters, thereby resulting in low available CoA levels and metabolic collapse including hypoglycemia^25,45^. NAD^+^ has been demonstrated to attenuate or prevent hepatosteatosis and steatohepatitis, increase oxidative metabolism, stimulate mitochondrial biogenesis and prevent mitochondrial stress^21–23,46^. Notably, computational modelling of hepatic mFAO revealed a potential vicious cycle of CoA ester accumulation, CoASH depletion, and mFAO flux decline, which was aggravated by loss of MCAD activity^47^. In the underlying mechanism the CoASH-consuming enzyme medium-chain ketoacyl-CoA thiolase (MCKAT) and the NAD^+^-reducing enzyme medium/short-chain hydroxyacyl-CoA dehydrogenase played a key role^48^. Upregulation of MCKAT and of the mitochondrial [NAD^+^]-[NADH] ratio substantially alleviated this phenotype^48^. This is in agreement with experimental findings that activation of MCKAT by downregulation of its inhibitor p46Shc^49^ and upregulation of NAD^+ ^^50^ stimulate the mFAO flux. Upregulation of the mitochondrial thioesterase (*Acot2*) would lead to enhanced hydrolysis of mitochondrial acyl-CoAs and thereby make free CoA available. Computational modeling suggested that this could stimulate the fatty-acid oxidation under conditions of excessive sequestration of CoA in acyl-CoA esters (not shown). Common transcriptional regulatory mechanisms for genes involved in CoASH- and NAD(P)(H) metabolism are still unknown, as far as we are aware.

A limitation of this study is that biochemical evidence for a role of CoASH and NAD(P)(H) metabolism in the adaptation to loss of MCAD activity was not exhaustively investigated. We have however, as a proof of principle, confirmed differential expression of *Acot2*. ACOT2 is a mitochondrial thioesterase^30,32^ that produces free (non-esterified) CoA. This may serve as a substrate for the mFAO enzymes CPT2 and the abovementioned MCKAT and thereby stimulate mFAO in the absence of MCAD activity. In future studies we expect to study the roles of CoASH and NAD(P)(H) metabolism in the robustness of the MCAD-KO mouse.

## Materials and Methods

### Animals, Experimental design and Tissue sampling

Male MCAD-KO and littermate wild-type (WT) mice on a C57BL/6J background (backcrossed for 10 generations) were housed in a temperature- (21 °C) and light-controlled (12 hrs light) facility and fed commercially available laboratory chow (ABDiets, Woerden, The Netherlands). Mice used in the experimental procedures were 2–4 months of age. All experiments were approved by the Ethics Committee for Animal Experiments of the University of Groningen. From the start of the experiment, mice were fed a low-fat or high-fat semi-synthetic diet (as in D12450B or D12451^51^, Research Diet Services, Wijk Bij Duurstede, The Netherlands, respectively) for 6 weeks, and had free access to drinking water. The high-fat diet contained 45% of calories from palm oil fat, in order to mimic the ratio of saturated to monounsaturated to polyunsaturated fatty acids (40:40:20) in a human diet. Mice were subsequently either fasted for 16 hrs (denoted as ‘fasted mice’) or kept on the diet for these 16 hours (denoted as ‘fed mice’). The food was removed from the fasting mice between 5 and 7 pm, i.e. 0–2 hours before the light was switched off. Blood glucose concentrations were measured during the experiment using a OneTouch UltraEasy® monitor (LifeScan Benelux, Beerse, Belgium). All mice were terminated 16 hours later between 9 and 11 am by cardiac puncture under isoflurane anesthesia. Isoflurane treatment was kept as short as possible (typically less than 3 minutes), which should avoid secondary effects on metabolism^52,53^. After termination the liver was removed quickly, weighed, a piece of the liver freeze-clamped for further analysis, a piece of the liver was immersed in 10% buffered formalin and another piece was fixed and frozen in Tissue-Tek® fixative. Finally, a piece of the left large lobe of the liver was collected in a buffer containing 250 mM sucrose and 10 mM Tris (pH 7.0) and used directly for the isolation of fresh mitochondria. All methods were performed in accordance with the relevant guidelines and regulations.

### Liver histology

Pieces of livers that were fixed in formalin, were processed for paraffin sectioning, and stained with hematoxylin and eosin. Frozen liver sections that were thus fixed in Tissue-Tek® fixative were prepared using standard methods and sections were stained with Oil-Red-O. Slides were examined without knowledge of the diet. The slides were analyzed by board-certified veterinary pathologists, who scored the slides for nonalcoholic fatty liver disease (NAFLD) and NAFLD activity score (NAS) according to Kleiner *et al*.^54^.

### Hepatic triglyceride (TG) concentration

Freeze-clamped liver was crushed in liquid nitrogen and 15% (w/v) homogenates in PBS (pH 7.4) were prepared. Total liver lipids were extracted according to Bligh & Dyer^55^. The extracted lipids were redissolved in 2% Triton-X100 and measured using a commercially available kit for triglyceride (Roche Diagnostics, Mannheim, Germany) according to the manufacturer’s instructions.

### Oxygen consumption rates in fresh liver mitochondria

Mitochondria were isolated from fresh liver tissue according to Mildaziene *et al*.^56^, with the last three centrifugation steps at 800, 7200 and 7200 g instead of 750, 7000 and 7000 g, respectively. Pyruvate-, palmitoyl-CoA (C16-acyl-CoA)- and octanoyl-CoA (C8-acyl-CoA)-dependent oxygen consumption rates in these mitochondria were measured at 37 °C using a two-channel high-resolution Oroboros oxygraph-2k (Oroboros, Innsbruck, Austria) with malate-containing MiR05 buffer (i.e. mitochondrial respiration medium in Table 1 in ref.^57^). To measure the maximal ADP-stimulated oxygen consumption (state 3), 4.8 U ml^−1^ hexokinase, 12.5 mM glucose and 1 mM ATP was added. To measure the resting state oxygen consumption rate (state 4), ADP transport was blocked with 1.25 μM carboxyatractyloside.

### Targeted quantitative proteomics of mitochondrial proteins

A set of >50 mitochondrial proteins involved in substrate transport, oxidative phosphorylation (OXPHOS), mitochondrial fatty-acid β-oxidation, tricarboxylic acid cycle, and antioxidant activity were quantified in isolated mitochondria using isotopically labelled standards (^13^C-labeled lysines and arginines) derived from synthetic protein concatemers (QconCAT) (PolyQuant GmbH, Bad Abbach, Germany) according to Wolters *et al*.^28^.

### RNA isolation, RNASeq analysis and quantitative reverse transcriptase polymerase chain (qRT-PCR)

A description of the RNA isolation, RNASeq analysis and qRT-PCR methods is given in Supplementary Text 1.

### Origin of mixed-background MCAD-KO mice microarray data

Previously published microarray data^27^ (GEO accession number GSE37546) that were used for pattern recognition analysis originate from livers of C57BL/6NTac & 129P2/OlaHsd mixed-background MCAD-KO and littermate wild-type mice. Only microarray data from conditions comparable to those in this study were used, i.e. from mice that were injected with saline and subsequently fasted for 12 hours.

### Pattern recognition on RNAseq and microarray data

#### Gene-set enrichment analysis

Gene-set enrichment analysis (GSEA) was performed according to Subramanian *et al*. 2005^58^ using the GSEA desktop Java application software (version 2.2.4, Broad Institute). Per WT-KO comparison, pre-ranked gene lists containing log2-counts-per-million t-value, together with the RNASeq chip file and the gene set collection of interest were used as input for the application. For the GSEA of the microarray data from studies with the mixed-background mice, the pre-ranked gene lists that contained the Limma t-value^59^ and the.chip file that corresponded to the arrays that were used in the mixed-background studies^60^ were used (mouse4302mmentrezg.chip). GSEA was done for a metabolic gene set collection that consisted of the KEGG metabolic gene set collection (downloaded in R – Version 6.0), complemented with three newly defined gene sets. These gene sets contained genes coding for (i) enzymes that catalyze reactions involving Coenzyme A (CoA gene set), (ii) all reactions involving NAD(P)^+^ and NAD(P)H (NAD(P)(H) gene set), and (iii) enzymes catalyzing only the redox reactions involving NAD(P)(H) (NAD(P)(H) redox gene set) and not, for instance, cofactor biosynthesis reactions. The GSEA desktop Java application software was set to perform 1000 permutations and use weighed enrichment statistic. Furthermore, for each gene set with a false discovery rate (FDR) of <25%, it was also calculated whether CoA and NAD(P)(H) genes were overrepresented in the contributing genes list. This was done by calculating a risk ratio (RR) parameter according to

$$R{R}_{CoA\_NADPH}=\frac{\#C{G}_{CoA\_NADPH}/\#CG}{(\#gene{s}_{CoA\_NADPH}-\#C{G}_{CoA\_NADPH})/(\#genes-\#CG)}$$

and determining whether this value is larger than 1. Here #CG is the number of contributing genes in the gene set of interest, #CG_CoA_NADPH_ is the number of contributing genes in this gene set that are also CoA or NAD(P)(H) genes, #genes is the total number of genes in the gene set and #genes_CoA_NADPH_ is the total number of CoA and NAD(P)(H) genes in the gene set.

#### Alternative gene set enrichment analysis

As an alternative for the classical Gene Set Enrichment Analysis, a list of differentially-expressed genes was made for each WT vs KO comparison, based on the following inclusion criteria: (|log2(fold change)| > 0.5 AND p < 0.1) OR p < 0.05. This represents a compromise between statistical significance (low p value) and biological relevance (large change). We generated separate lists for up and downregulated genes and we generated lists from both the DeSeq2 differential gene expression (DGE) results and the Voom DGE results. We then calculated for each WT-KO comparison, for each metabolic gene set, and for each direction separately, what percentage of the differentially-expressed genes were present in the gene set and the gene sets were subsequently ranked according to this percentage. Gene sets with the same percentage received the same ranking and gene sets with a percentage of zero received the rank corresponding to the number of metabolic gene sets in the metabolic gene set collection. The ranked gene sets were subsequently given an overall rank based on the median of the ranks of the conditions. Furthermore, for each condition, for both up and downregulation, it was determined whether the number of differentially expressed genes of the given gene set is overrepresented in the list of differentially-expressed genes. This was calculated by determining whether the corresponding risk-ratio (RR_gene set_) is larger than 1, according to:

$$R{R}_{geneset}=\frac{\#DE{G}_{metabolic,geneset}/\#DE{G}_{metabolic,all}}{(\#gene{s}_{geneset}-\#DE{G}_{metabolic,geneset})/(\#metabolic\,genes-\#DE{G}_{metabolic,all})}$$

where #DEG_metabolic, all_ is the number of differentially expressed metabolic genes, #DEG_metabolic, gene set_ is the number of differentially expressed metabolic genes belonging to the gene set in question, “#genes_gene set_” is the total number of genes in the gene set and “#metabolic genes” is the total number of metabolic genes, according to the metabolic gene set collection.

### Oxidative damage in plasma

Thiobarbituric acid reactive species (TBA-RS), a measure for malondialdehyde, a parameter of lipid oxidative damage, were determined spectrophotometrically in plasma as performed by Derks *et al*.^44^.

### Hepatic citrate synthase activity

The hepatic citrate synthase activity was determined as performed by Stolle *et al*.^61^.

### Statistical analysis

Differences in Body weight (BW), Blood glucose (BG), liver weight (LW) and oxygen consumption were assessed with ANOVA and post-hoc LSD after log transformation if the data was not normally-distributed. Proteomic data were analyzed by Kruskal-Wallis ANOVA and Mann Whitney U test. *Ppia*-normalized Acot2 mRNA expression data generated by qRT-PCR were assessed for normality and subsequently the differences between WT and KO was assessed by Student’s t-test. These analyses were performed using IBM SPSS Statistics version 22.0 (SPSS Inc., Chicago, IL, USA. The level of significance was set at p < 0.05.

## Supplementary information

Supplementary figures and supplementary text

Supplementary Tables

## Data Availability

The transcriptomic dataset generated during the current study are available on the NCB Geo Profiles repository (GEO Accession Number GSE136309).
