# Supplementary material for: Transcriptome analysis suggests a compensatory role of the cofactors coenzyme A and NAD+ in medium-chain acyl-CoA dehydrogenase knockout mice
Source: Sci Rep. 2019 Oct 10;9:14539. doi: 10.1038/s41598-019-50758-0 (PMC6787083; doi:10.1038/s41598-019-50758-0)
Supplement: Supplementary file 1 — Supplementary figures and supplementary text [file 41598_2019_50758_MOESM1_ESM.pdf]

Transcriptome analysis suggests a compensatory role of the cofactors coenzyme A and NAD^+ in medium-chain acyl-CoA dehydrogenase knockout mice

Authors:

Anne-Claire M.F. Martines, Albert Gerding, Sarah Stolle, Marcel A. Vieira-Lara, Justina C. Wolters, Angelika Jurdzinski, Laura Bongiovanni, Alain de Bruin, Pieter van der Vlies, Gerben van der Vries, Vincent W. Bloks, Terry G.J. Derks, Dirk-Jan Reijngoud, Barbara M. Bakker

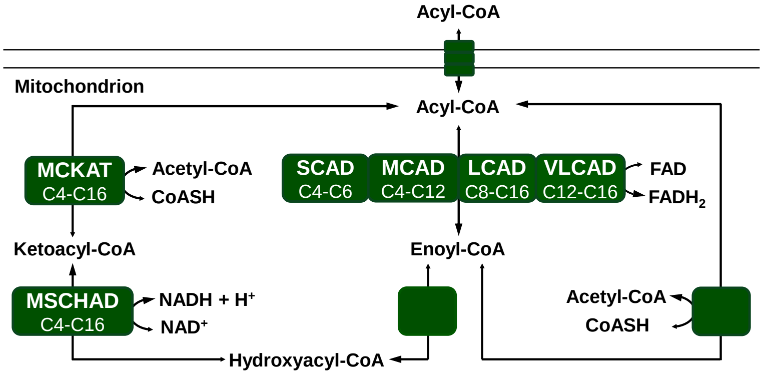

Supplementary Figure 1. Schematic representation of the murine mFAO pathway. CoASH: free Coenzyme A; Boxes represent individual enzymes. Only enzymes that are mentioned in the main text are specifically portrayed with a name and chain length specificity; VLCAD, LCAD, MCAD and SCAD: very-long-, long-, medium-, and short-chain acyl-CoA dehydrogenase, respectively; MCKAT: medium-chain ketoacyl-CoA thiolase; MSCHAD: medium/short-chain hydroxyacyl-CoA dehydrogenase. This figure was created by the first author.

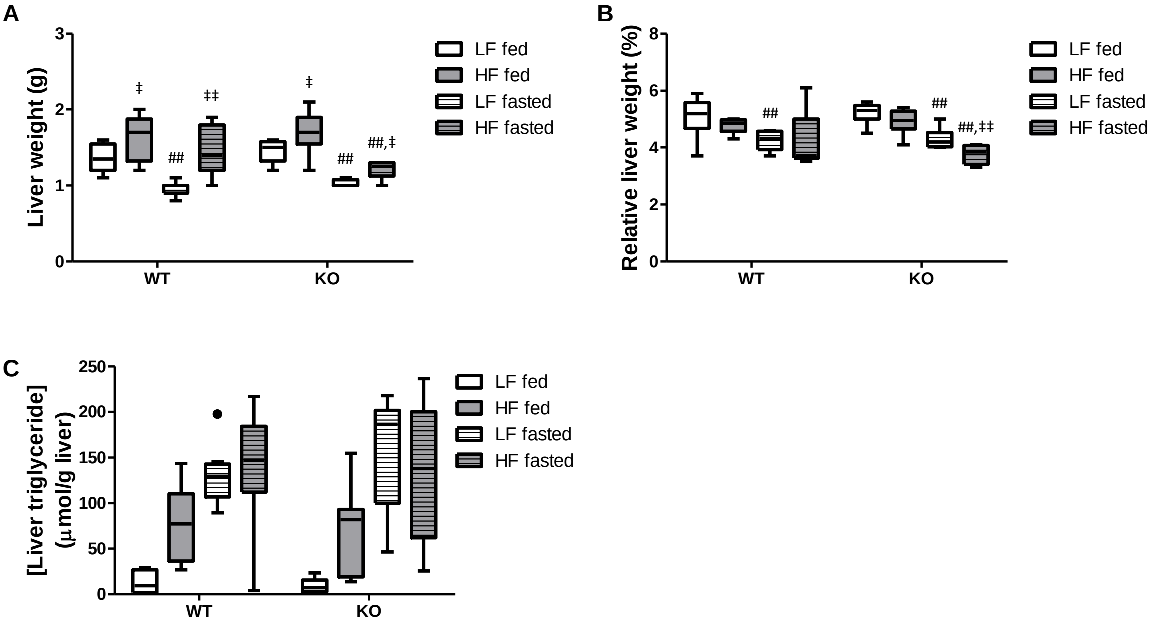

Supplementary Figure S2. Liver weight (A), relative liver weight (B) and liver triglyceride content (C). The results are represented as Tukey box and whisker plots, where the black-filled jagged circles indicate individual mice falling outside the 75% percentile plus 1.5·inter-quartile range (IQR) or 25% percentile minus 1.5·IQR. n=6-8 for both WT and KO. *: $p<0.05$ compared to WT, # and ##: $p<0.05$ and $p<0.01$ compared to fed, respectively, ‡ and ‡‡: $p<0.05$ and $p<0.01$ compared to low-fat diet (LF), respectively.

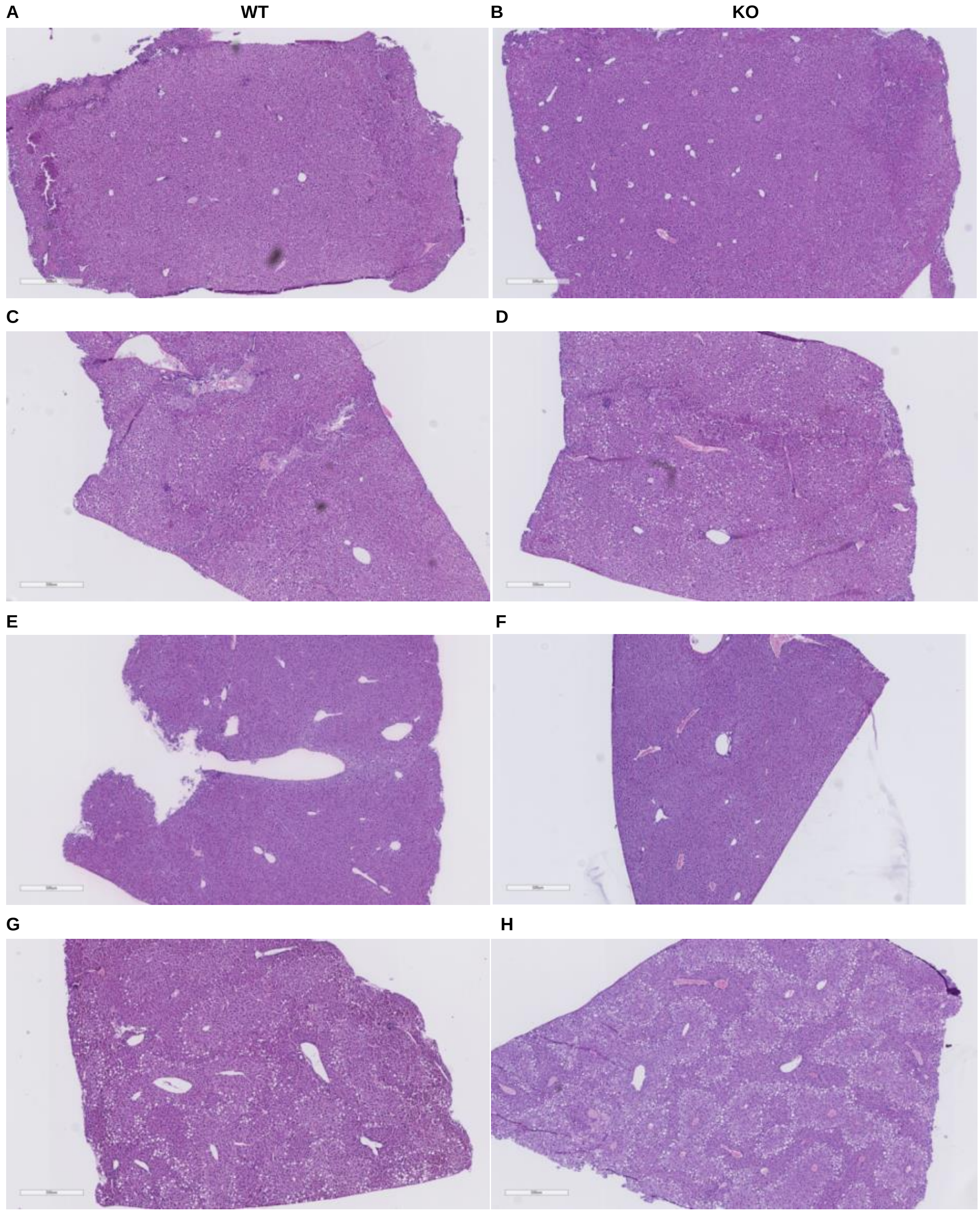

Supplementary Figure S3. Liver tissue integrity determined by H&E staining. A-B: Low-fat fed, C-D, High-fat fed, E-F: Low-fat fasted, G-H: High-fat fasted. A, C, E, G: WT. B,D,F, G: KO. Shown here are representative pictures at 1x magnification

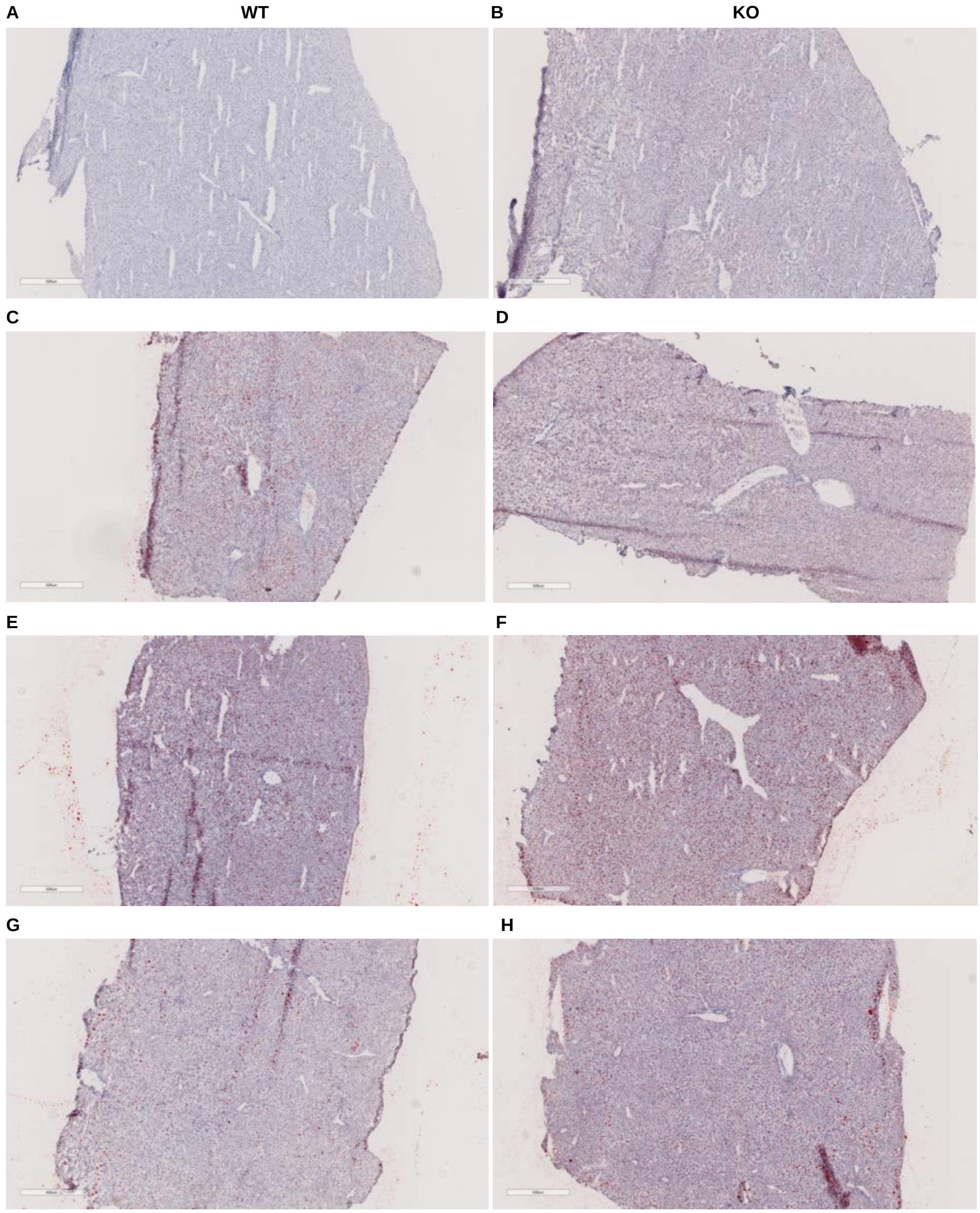

Supplementary Figure S4. Extent of steatosis determined by Oil Red O staining. A-B: Low-fat fed, C-D, High-fat fed, E-F: Low-fat fasted, G-H: High-fat fasted. A, C, E, G: WT. B,D,F, G: KO. Shown here are representative pictures at 1x magnification

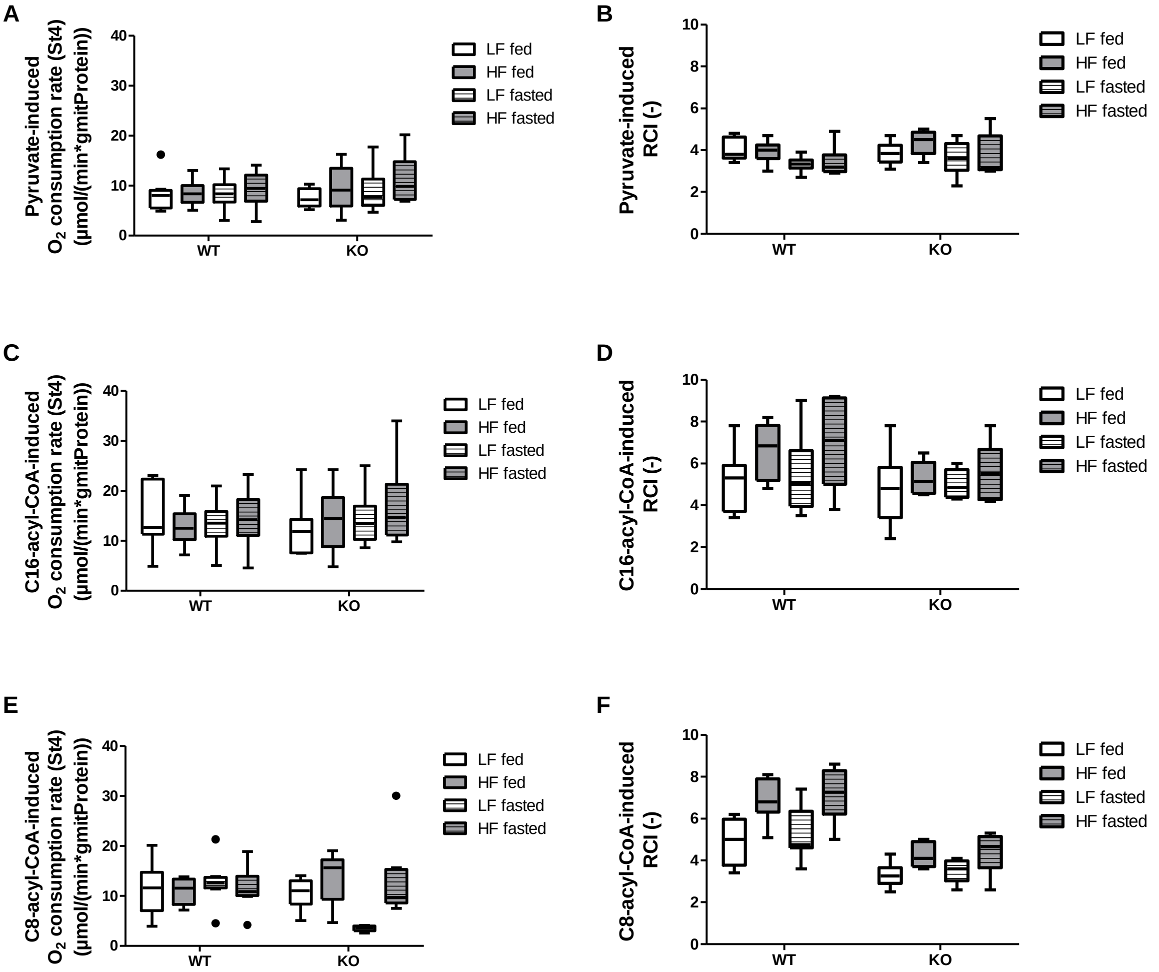

Supplementary Figure S5. Pyruvate- (A-B), C16-acyl-CoA – (C-D) and C8-acyl-CoA-induced (E-F) state 4 oxygen consumption flux (A,C,E) and RCI (B,D,F) in liver mitochondria. State 4 is the basal oxygen consumption. RCI is the respiratory control index, calculated as State 3 O_2 consumption rate divided by State 4 O_2 consumption rate. The results are represented as Tukey box and whisker plots, where the black-filled jagged circles indicate individual mice falling outside the 75% percentile plus 1.5·inter-quartile range (IQR) or 25% percentile minus 1.5·IQR.n=6 for both WT and KO. LF: Low-fat, HF: High-fat, RCI: Respiratory control index; this is calculated by dividing the O_2 consumption rate in State 3 by the O_2 consumption rate in State 4.

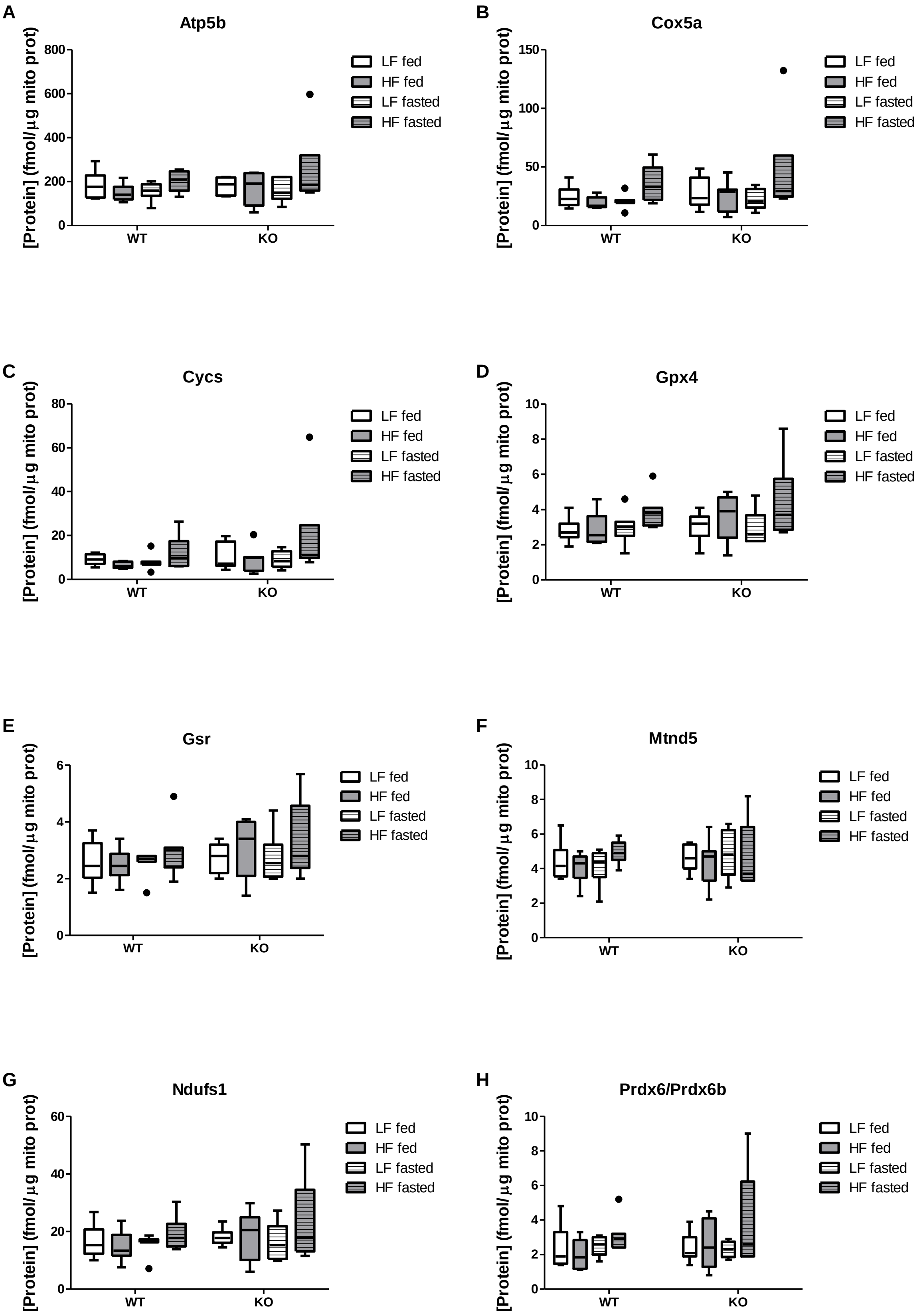
A
Atp5b
[Protein] (fmol/µg mito prot)
800
600
400
200
0
WT
KO
LF fed
HF fed
LF fasted
HF fasted
B
Cox5a
[Protein] (fmol/µg mito prot)
150
100
50
0
WT
KO
LF fed
HF fed
LF fasted
HF fasted
C
Cycs
[Protein] (fmol/µg mito prot)
80
60
40
20
0
WT
KO
LF fed
HF fed
LF fasted
HF fasted
D
Gpx4
[Protein] (fmol/µg mito prot)
10
8
6
4
2
0
WT
KO
LF fed
HF fed
LF fasted
HF fasted
E
Gsr
[Protein] (fmol/µg mito prot)
6
4
2
0
WT
KO
LF fed
HF fed
LF fasted
HF fasted
F
Mtnd5
[Protein] (fmol/µg mito prot)
10
8
6
4
2
0
WT
KO
LF fed
HF fed
LF fasted
HF fasted
G
Ndufs1
[Protein] (fmol/µg mito prot)
60
40
20
0
WT
KO
LF fed
HF fed
LF fasted
HF fasted
H
Prdx6/Prdx6b
[Protein] (fmol/µg mito prot)
10
8
6
4
2
0
WT
KO
LF fed
HF fed
LF fasted
HF fasted

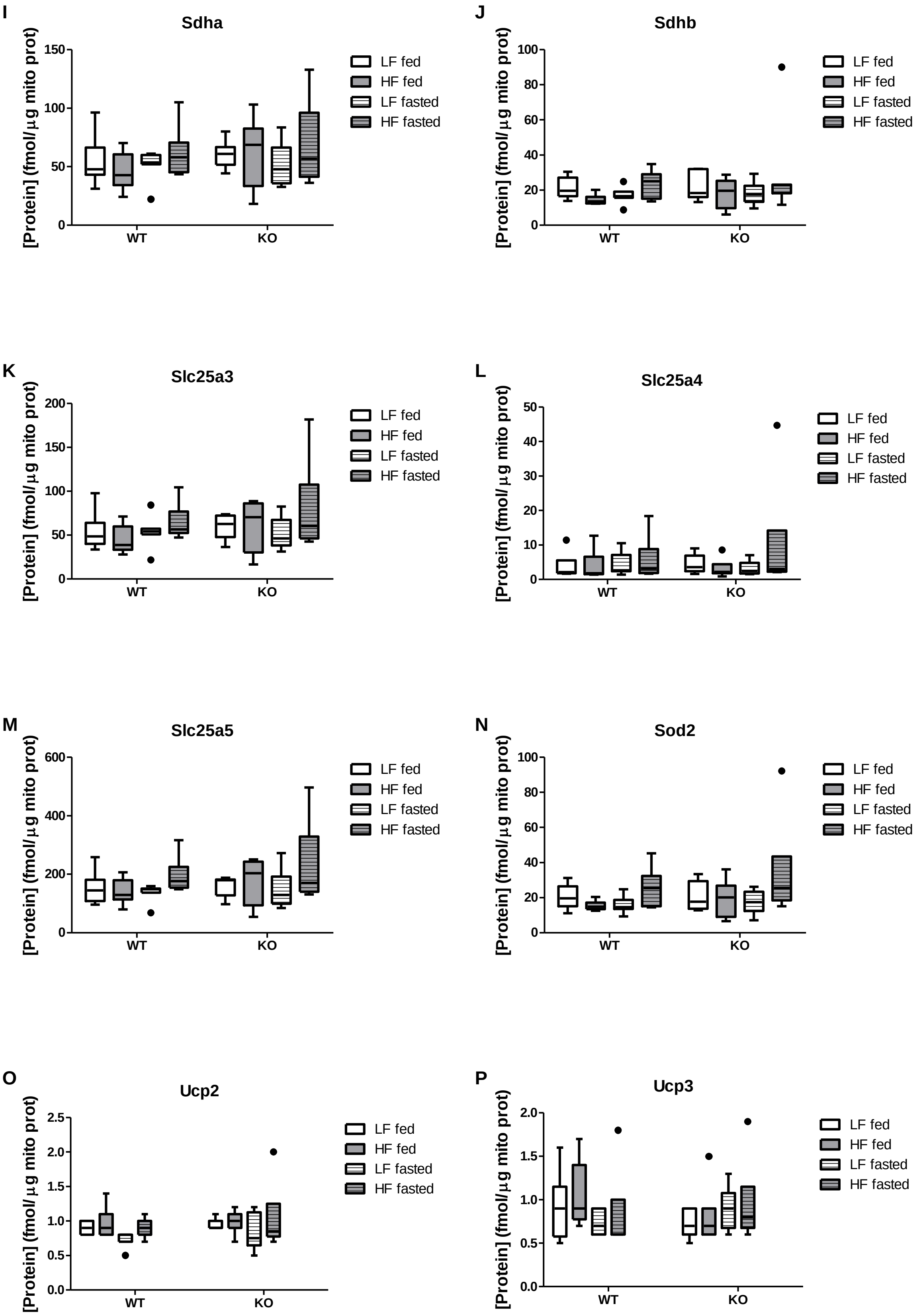
I
Sdha
J
Sdhb
K
Slc25a3
L
Slc25a4
M
Slc25a5
N
Sod2
O
Ucp2
P
Ucp3
[Protein] (fmol/µg mito prot)
WT
KO
LF fed
HF fed
LF fasted
HF fasted

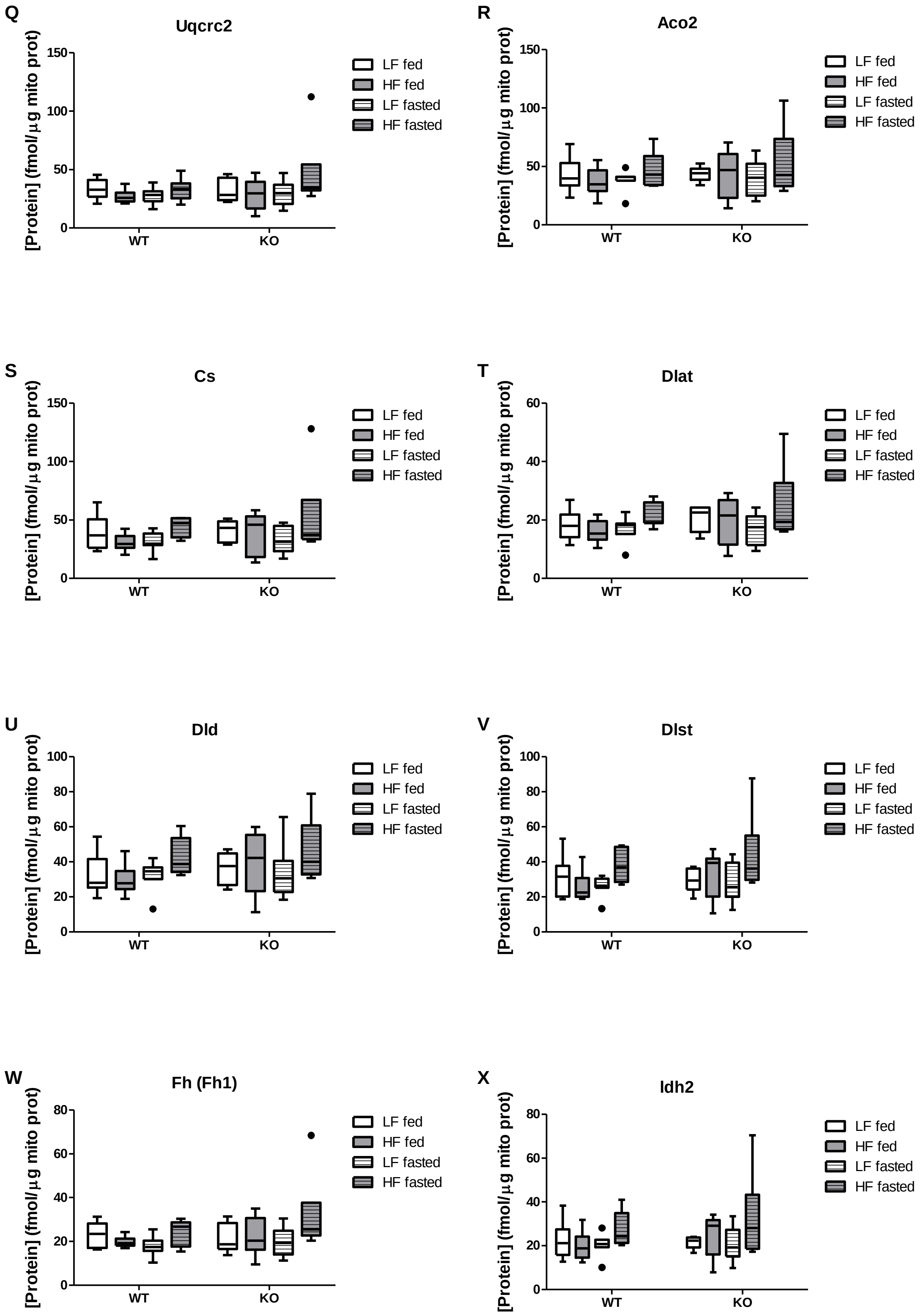
Q
Uqcrc2
R
Aco2
S
Cs
T
Dlat
U
Dld
V
Dlst
W
Fh (Fh1)
X
Idh2
[Protein] (fmol/µg mito prot)
WT
KO
LF fed
HF fed
LF fasted
HF fasted

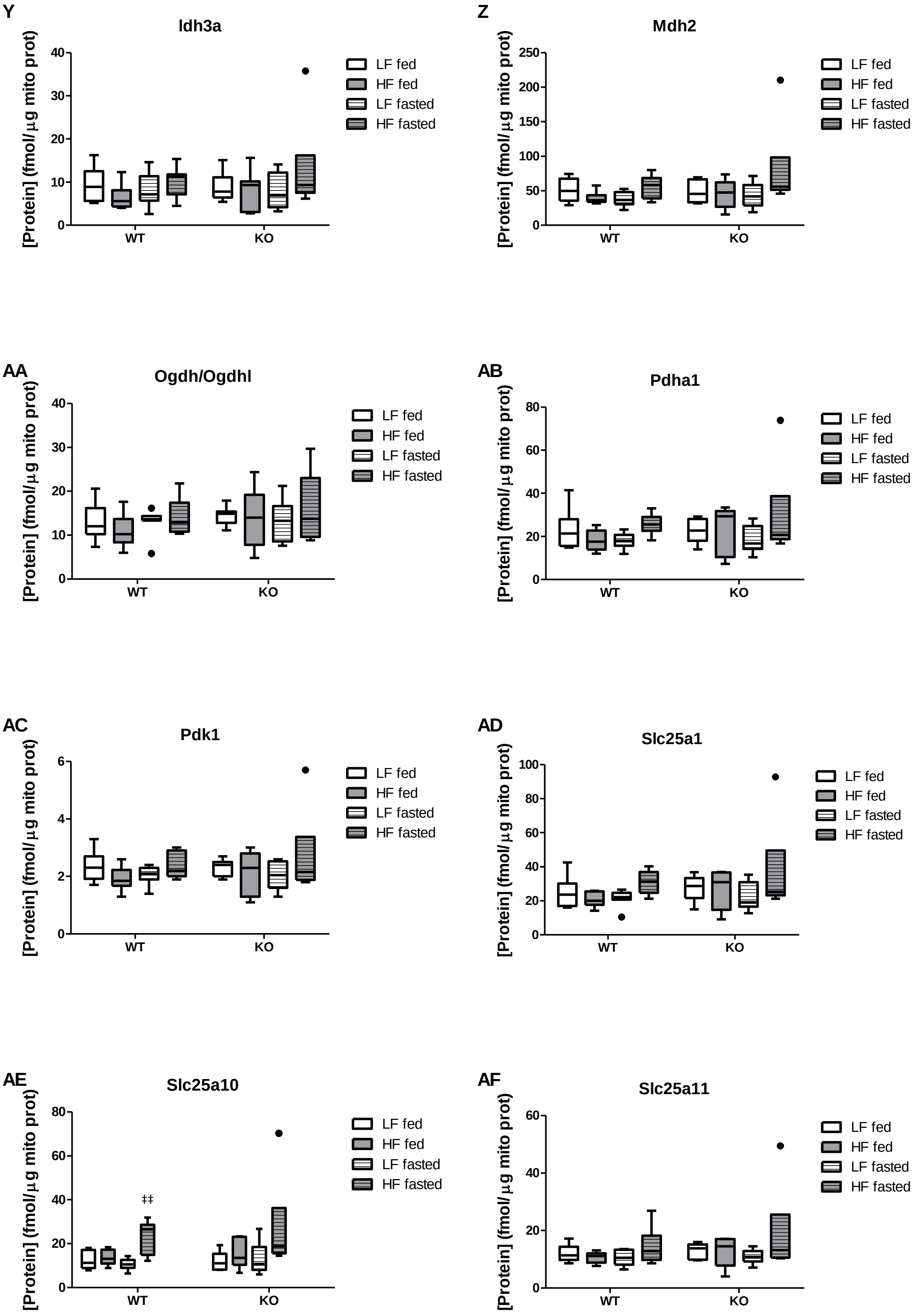
Y
Idh3a
[Protein] (fmol/µg mito prot)
0
10
20
30
40
WT
KO
LF fed
HF fed
LF fasted
HF fasted
Z
Mdh2
[Protein] (fmol/µg mito prot)
0
50
100
150
200
250
WT
KO
LF fed
HF fed
LF fasted
HF fasted
AA
Ogdh/Ogdhl
[Protein] (fmol/µg mito prot)
0
10
20
30
40
WT
KO
LF fed
HF fed
LF fasted
HF fasted
AB
Pdha1
[Protein] (fmol/µg mito prot)
0
20
40
60
80
WT
KO
LF fed
HF fed
LF fasted
HF fasted
AC
Pdk1
[Protein] (fmol/µg mito prot)
0
2
4
6
WT
KO
LF fed
HF fed
LF fasted
HF fasted
AD
Slc25a1
[Protein] (fmol/µg mito prot)
0
20
40
60
80
100
WT
KO
LF fed
HF fed
LF fasted
HF fasted
AE
Slc25a10
[Protein] (fmol/µg mito prot)
0
20
40
60
80
‡‡
WT
KO
LF fed
HF fed
LF fasted
HF fasted
AF
Slc25a11
[Protein] (fmol/µg mito prot)
0
20
40
60
WT
KO
LF fed
HF fed
LF fasted
HF fasted

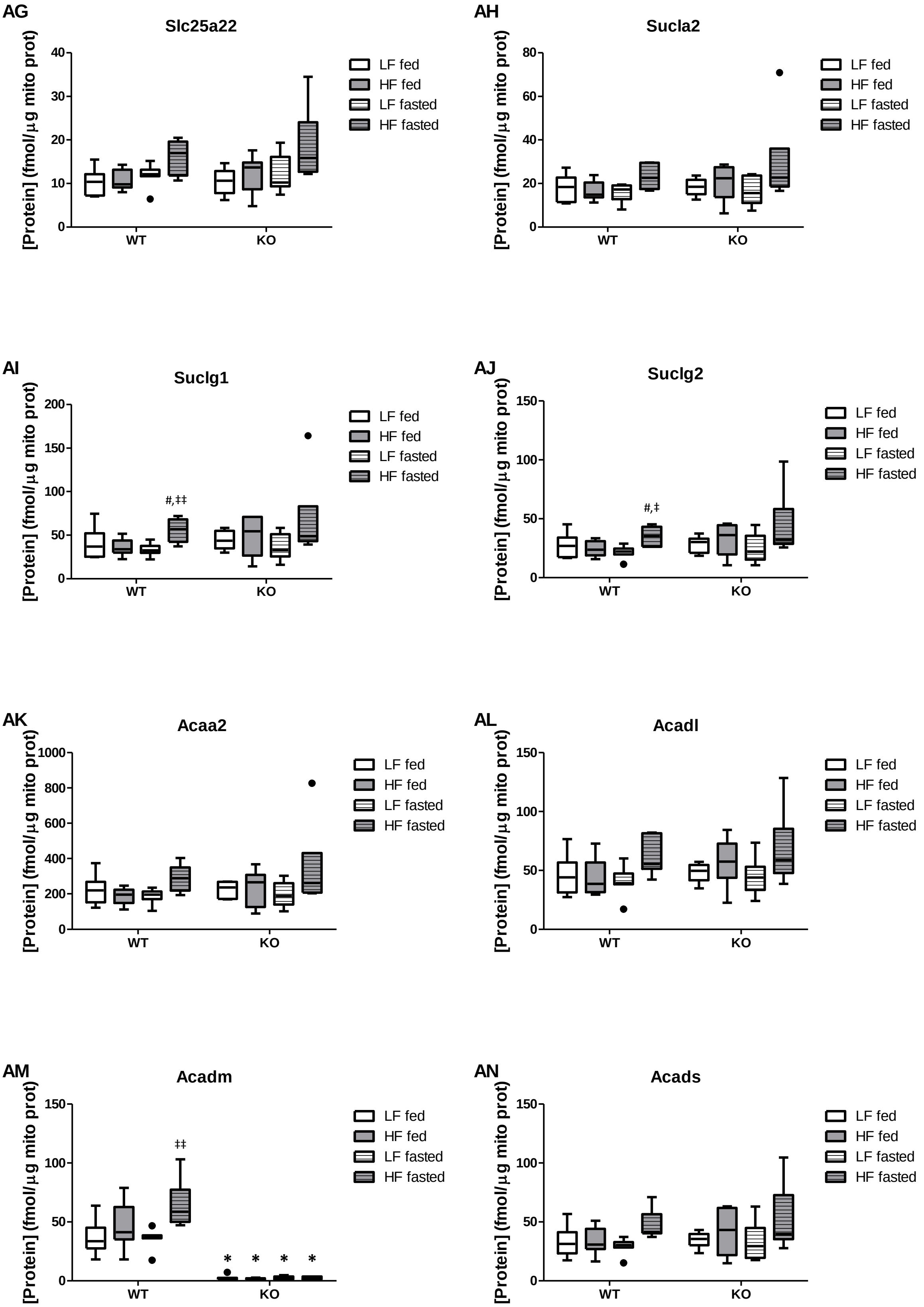
AG
Slc25a22
[Protein] (fmol/µg mito prot)
0
10
20
30
40
WT
KO
LF fed
HF fed
LF fasted
HF fasted
AH
Sucla2
[Protein] (fmol/µg mito prot)
0
20
40
60
80
WT
KO
LF fed
HF fed
LF fasted
HF fasted
AI
Suclg1
[Protein] (fmol/µg mito prot)
0
50
100
150
200
#,‡‡
WT
KO
LF fed
HF fed
LF fasted
HF fasted
AJ
Suclg2
[Protein] (fmol/µg mito prot)
0
50
100
150
#,‡
WT
KO
LF fed
HF fed
LF fasted
HF fasted
AK
Acaa2
[Protein] (fmol/µg mito prot)
0
200
400
600
800
1000
WT
KO
LF fed
HF fed
LF fasted
HF fasted
AL
Acadl
[Protein] (fmol/µg mito prot)
0
50
100
150
WT
KO
LF fed
HF fed
LF fasted
HF fasted
AM
Acadm
[Protein] (fmol/µg mito prot)
0
50
100
150
‡‡
* * * *
WT
KO
LF fed
HF fed
LF fasted
HF fasted
AN
Acads
[Protein] (fmol/µg mito prot)
0
50
100
150
WT
KO
LF fed
HF fed
LF fasted
HF fasted

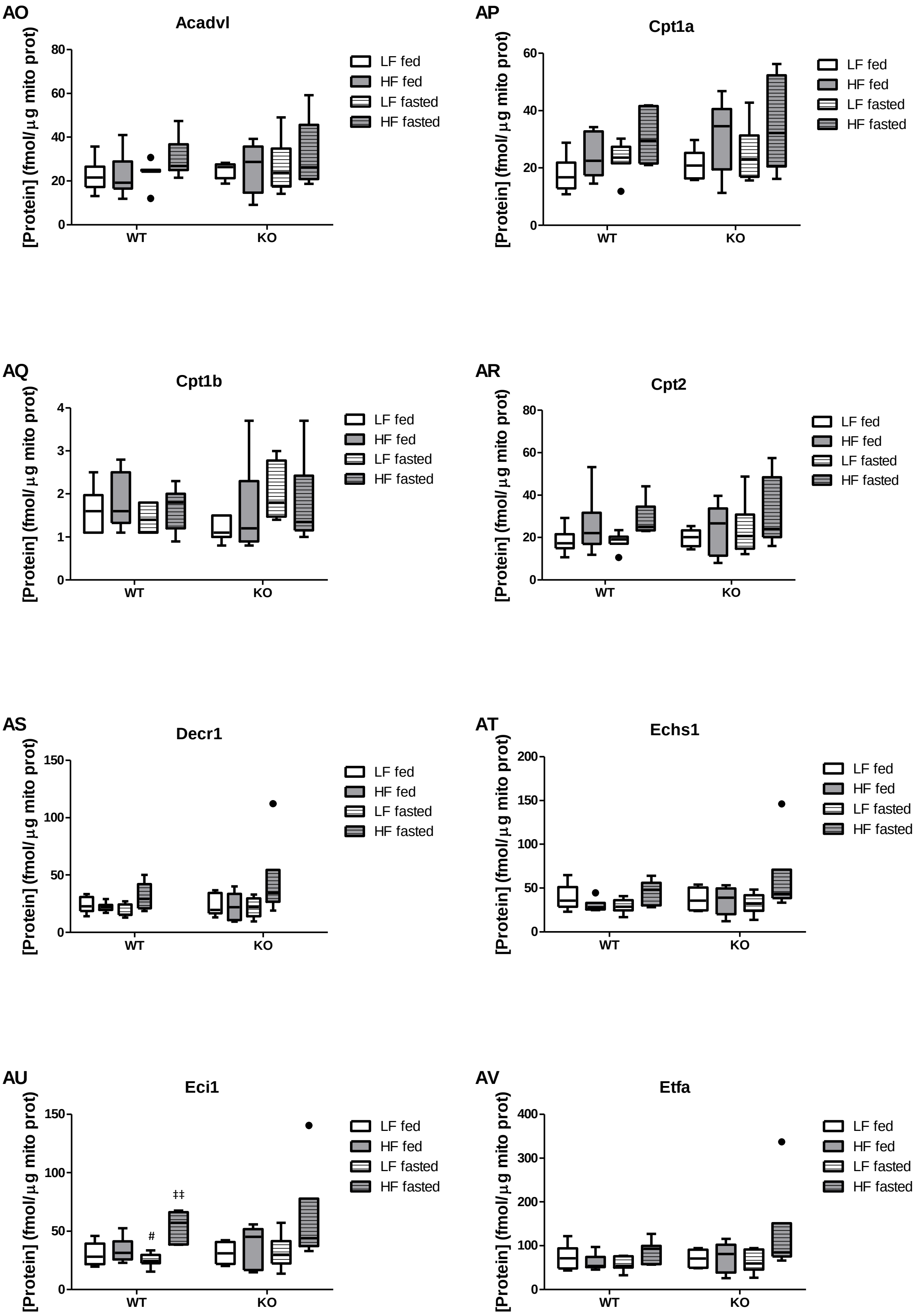
AO
Acadvl
AP
Cpt1a
AQ
Cpt1b
AR
Cpt2
AS
Decr1
AT
Echs1
AU
Eci1
AV
Etfa
[Protein] (fmol/µg mito prot)
WT
KO
LF fed
HF fed
LF fasted
HF fasted
#
‡‡

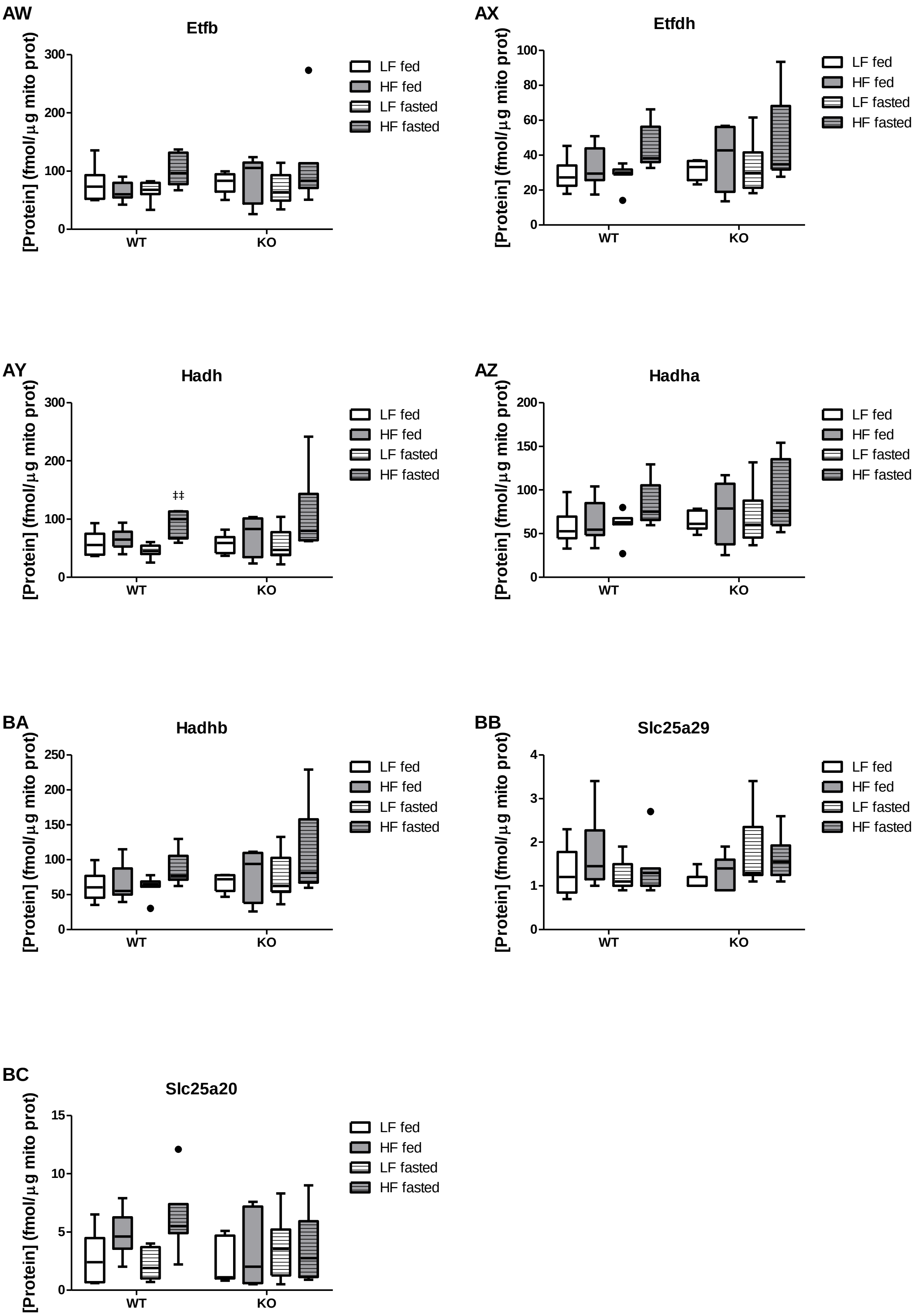

Supplementary Figure S6. Absolute levels of mitochondrial liver proteins. In the figure, the gene names corresponding to the proteins are given. The results are represented as Tukey box and whisker plots, where the black-filled jagged circles indicate individual mice falling outside the 75% percentile plus 1.5·inter-quartile range (IQR) or 25% percentile minus 1.5·IQR. LF: Low-fat HF: High-fat. n=6-8 for both WT and KO. *: $p<0.05$ compared to WT, # and ##: p<0. 05 and $p<0.01$ compared to fed, respectively, ‡ and ‡‡: $p<0.05$ and $p<0.01$ compared to LF, respectively.

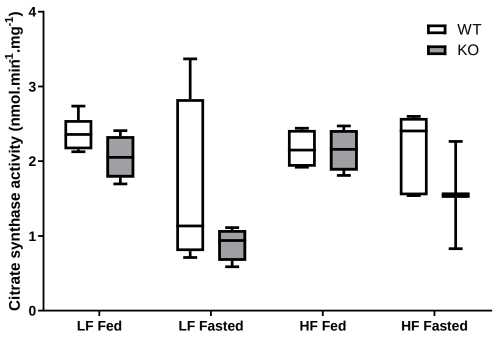

Supplementary figure S7. Citrate synthase activity in the liver of mice under the LF fed, LF fasted, HF fed and HF fasted conditions. The results are represented as Tukey box and whisker plots, where the black-filled squares indicate individual mice falling outside the 75% percentile plus 1.5·inter-quartile range (IQR) or 25% percentile minus 1.5·IQR. n=3-4 for WT and KO.

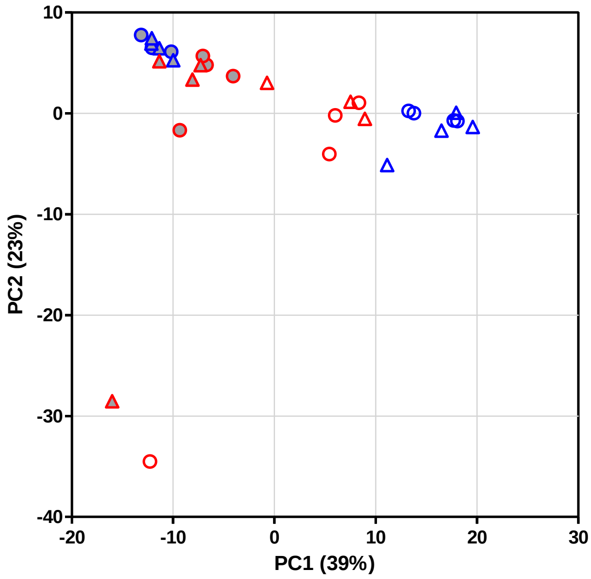

Supplementary figure S8. Principal Component Analysis of mRNA expression data. Grey-filled: Fed; White-filled: fasted; Triangle: KO, circle: WT; Blue: Low-fat diet; Red: High-fat diet. n=3-4 for WT and KO.

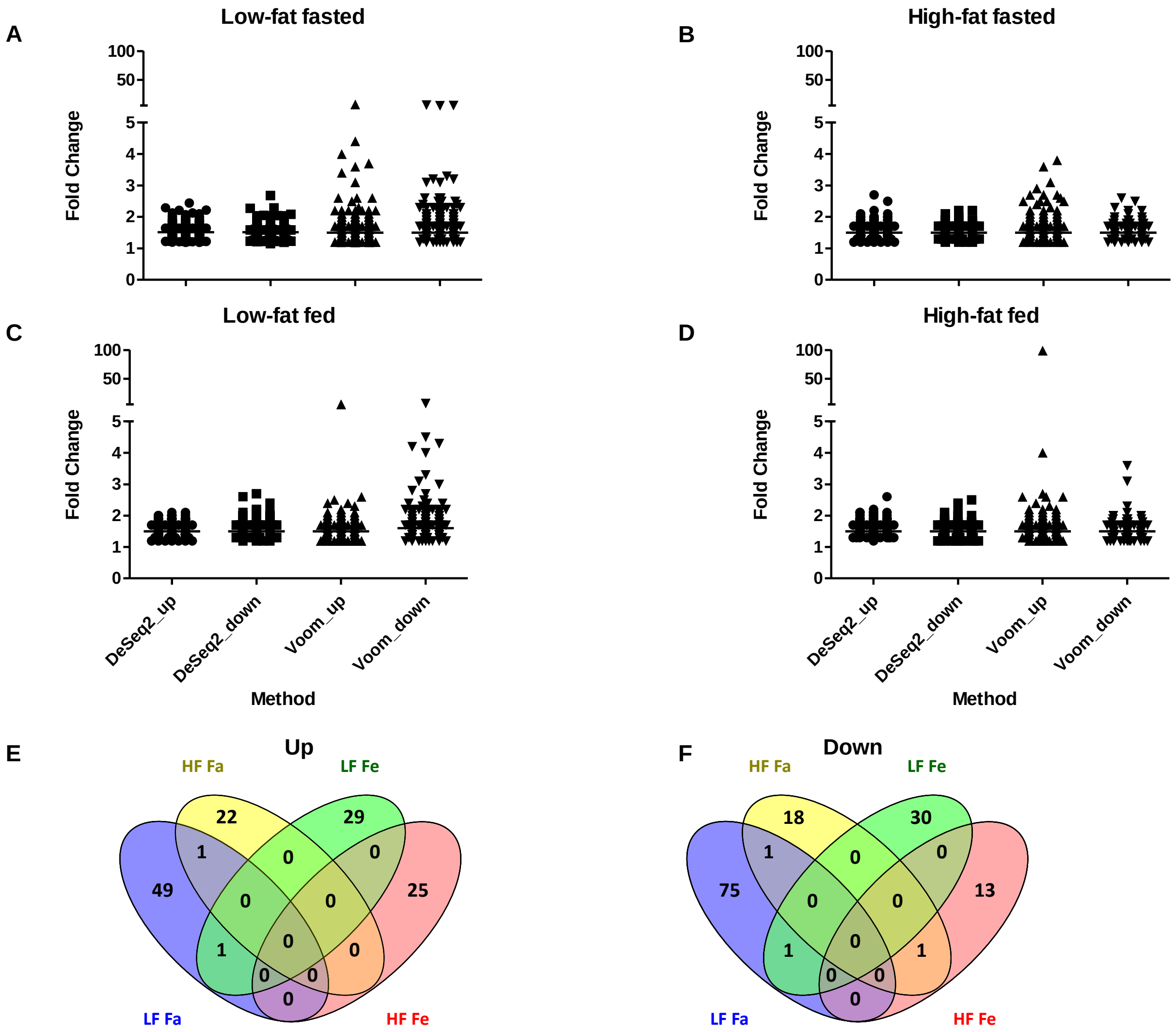

Supplementary figure S9. KO vs WT fold changes for the differential gene expression analysis methods DeSeq2 and Voom (A-D) and Venn diagrams for the Voom method (E-F) (p<0.01): LF = Low-fat diet, HF = High-fat diet, Fa = Fasted, Fe= Fed. n=3-4 per group.

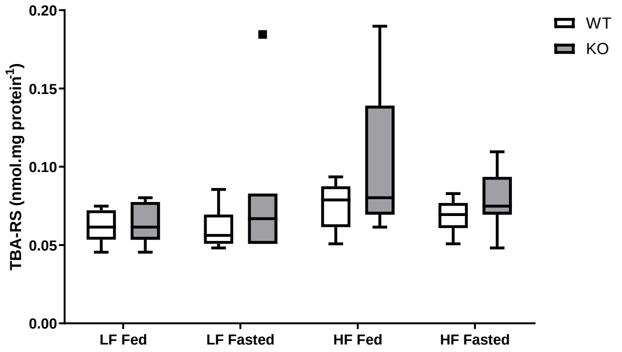

Supplementary figure S10. TBA-RS levels in plasma of mice under the LF fed, LF fasted, HF fed and HF fasted conditions. The results are represented as Tukey box and whisker plots, where the black-filled squares indicate individual mice falling outside the 75% percentile plus 1.5·inter-quartile range (IQR) or 25% percentile minus 1.5·IQR. n=7-8 for WT and.

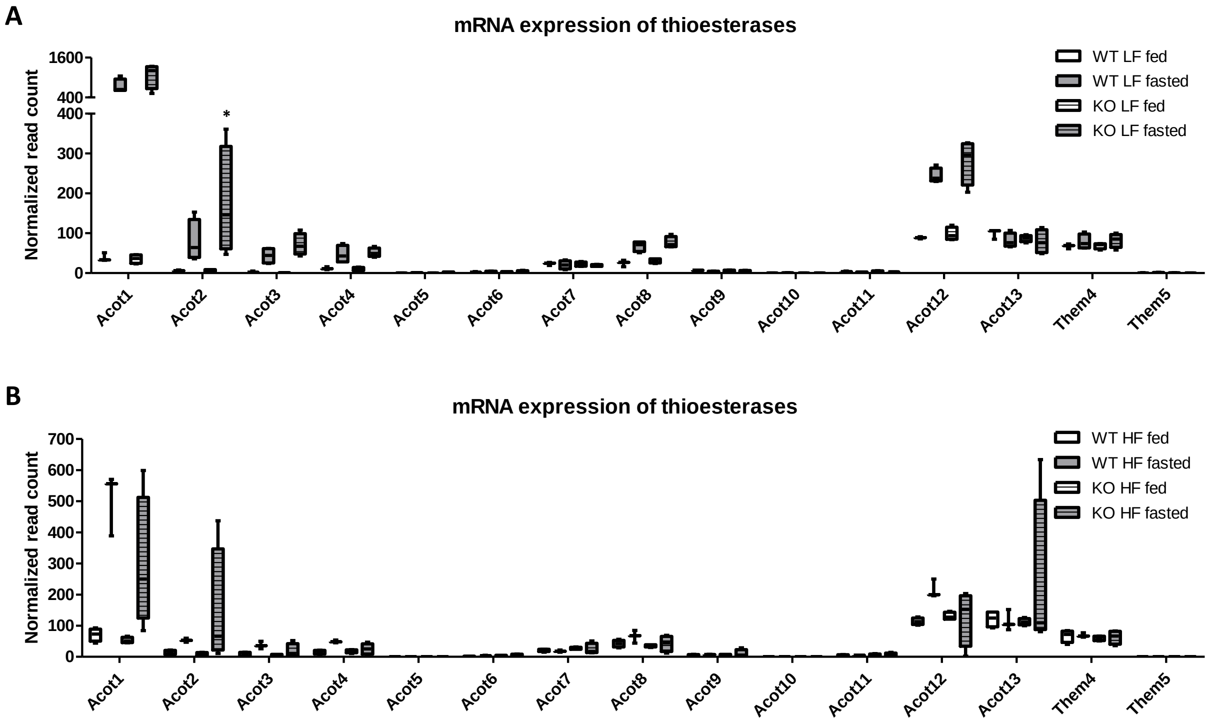

Supplementary figure S11. Acyl-CoA thioesterases gene expression in LF fed and LF fasted (A) and in HF fed and HF fasted (B) conditions. n=3 and 4 for WT and KO for high-fat (HF)- and low-fat (LF) conditions, respectively. The results are represented as Tukey box and whisker plots, where the black-filled jagged circles indicate individual mice falling outside the 75% percentile plus 1.5·inter-quartile range (IQR) or 25% percentile minus 1.5·IQR. n=6-8 for both WT and KO. *: p<0.05 compared to WT, # and ##: p<0. 05 and p<0.01 compared to fed, respectively, ‡ and ‡‡: p<0.05 and p<0.01 compared to low-fat (LF) diet, respectively.

Supplementary Text 1. RNA isolation, RNASeq analysis and quantitative reverse transcriptase polymerase chain (qRT-PCR)

RNA was isolated from crushed freeze-clamped liver with Trizol reagent (Invitrogen, Carlsbad, CA, USA). The purity and concentration of RNA was determined with NanoDrop ND-1000 (ISOGEN Life Science, De Meern, The Netherlands). Subsequently, RNA integrity and concentration were determined by capillary electrophoresis using PerkinElmer LabChipGX (PerkinElmer, Waltham, MA, USA). RNA samples with an integrity score above 7, a small 5S peak, and clearly distinguishable 18S and 28S peaks were used for subsequent sequencing analysis.

Sequence libraries were generated using the 3'QuantSeq sample preparation kits (Lexogen). The obtained cDNA fragment libraries were sequenced on an Illumina HiSeq2500 sequencer using default parameters (single read 1 x 50 bp) in pools of eight samples per lane. The obtained sequencing reads (fastQ files) were mapped to build Mus_musculus GRCm38 ensemble Release 82 reference genome using hisat/0.1.5-beta-goolf-1.7.20[1] with default settings. Subsequently, SAMtools/1.2- goolf-1.7.20 was used to sort the aligned reads[2]. Quantification of gene read counts was performed by HTSeq-count HTSeq/0.6.1p1 using –mode=union[3]. Analysis of differential gene expression (DGE) was performed using Voom transformation provided by the Limma package[4] and for comparison also with the DESeq2 package (version 1.10.1) in R (version 3.2.5), as indicated in the text. Principle component analysis (PCA) was performed on regularized log transformed raw counts[5] using the PCA function from the DESeq2 package (version 1.10.1) in R (version 3.2.5). For *Acot2* gene expression analysis by qRT-PCR, cDNA was synthesized from isolated liver RNA using Transcription Universal cDNA Master (Roche). mRNA expression was assayed by quantitative polymerase chain reaction using SYBR green (Roche) and the real-time polymerase chain reaction cycler (QuantStudio 7 Flex Real-Time PCR System, Applied Biosystems) using the primers AGTCAACGACGCAAAATGGTG (forward) and GCTCTTCCAATCCTGTTGGC (reverse) (Thermoscientific Invitrogen)[6]. *Acot2* gene expression was normalized against peptidyl-prolyl-cis-trans isomerase A (*Ppia*) using the primers TTCCTCCTTTCACAGAATTATTCCA (forward) and CCGCCAGTGCCATTATGG (reverse) (Thermoscientific Invitrogen). Expression data were analyzed using QuantStudio Real Time PCR software version 1.2 (Applied Biosystems) and the standard curve method of calculation. Results were presented as expression normalized to the expression of *Ppia* (coding for Peptidyl-prolyl cis-trans isomerase A, also known as Cyclophilin A)

References

1. Dobin, A. *et al.* STAR: Ultrafast universal RNA-seq aligner. *Bioinformatics* **29**, 15–21 (2013).
2. Li, H. *et al.* The Sequence Alignment/Map format and SAMtools. *Bioinformatics* **25**, 2078–2079 (2009).
3. Anders, S., Pyl, P. T. & Huber, W. HTSeq-A Python framework to work with high-throughput sequencing data. *Bioinformatics* **31**, 166–169 (2015).
4. Law, C. W., Chen, Y., Shi, W. & Smyth, G. K. Voom: Precision weights unlock linear model analysis tools for RNA-seq read counts. *Genome Biol.* **15**, 1–17 (2014).
5. Love, M. I., Huber, W. & Anders, S. Moderated estimation of fold change and dispersion for RNA-seq data with DESeq2. *Genome Biol.* **15**, 1–21 (2014).
6. Ellis, J. M., Bowman, C. E. & Wolfgang, M. J. Metabolic and Tissue-Specific Regulation of Acyl-CoA Metabolism. *PLoS One* **10**, e0116587 (2015).
